# Large scale genome reconstructions illuminate Wolbachia evolution

**DOI:** 10.1038/s41467-020-19016-0

**Published:** 2020-10-16

**Authors:** Matthias Scholz, Davide Albanese, Kieran Tuohy, Claudio Donati, Nicola Segata, Omar Rota-Stabelli

**Affiliations:** 1 Research and Innovation Centre, Fondazione Edmund Mach (FEM), San Michele all’Adige, Italy; 2 Department CIBIO, University of Trento, Trento, Italy; 3 Present Address: Centre Agriculture Food Environment (C3A), University of Trento, Trento, Italy

**Keywords:** Phylogenetics, Genomics, Microbiology, Parasite evolution

## Abstract

*Wolbachia* is an iconic example of a successful intracellular bacterium. Despite its importance as a manipulator of invertebrate biology, its evolutionary dynamics have been poorly studied from a genomic viewpoint. To expand the number of *Wolbachia* genomes, we screen over 30,000 publicly available shotgun DNA sequencing samples from 500 hosts. By assembling over 1000 *Wolbachia* genomes, we provide a substantial increase in host representation. Our phylogenies based on both core-genome and gene content provide a robust reference for future studies, support new strains in model organisms, and reveal recent horizontal transfers amongst distantly related hosts. We find various instances of gene function gains and losses in different super-groups and in cytoplasmic incompatibility inducing strains. Our *Wolbachia*-host co-phylogenies indicate that horizontal transmission is widespread at the host intraspecific level and that there is no support for a general *Wolbachia*-mitochondrial synchronous divergence.

## Abstract (web summary)

By greatly expanding the number of assembled genomes for *Wolbachia* (a group of intracellular bacteria) and constructing robust phylogenies, this study finds strong rate heterogeneity among *Wolbachia*populations and no support for synchronous divergence between *Wolbachia* and host mitochondria.

## Introduction

Nature is filled with exemplar cases of symbiotic interaction between bacteria and multicellular eukaryotes. While bacteria in such partnerships benefit from a protected environment, hosts are endowed with different fitness advantages such as key nutrients and antimicrobial protection^1–3^. The most studied and evolutionary remarkable case of symbiotic interaction is that of *Wolbachia*, an alpha-proteobacterium which infects animal cells^4^ and specifically invertebrates belonging to the Ecdysozoa, the superphylum encompassing nematodes, insects, and other arthropods^5^. *Wolbachia* infection is extremely widespread, and estimated to occur in up to 40–50% of terrestrial arthropods^6,7^, implying more than a million infected host species worldwide.

One peculiarity of *Wolbachia* is that it not only provides fitness advantages to the host by conferring for example microbial protection, but it can also manipulate the host reproductive biology to increase its own chances of transmission using for example sperm-egg incompatibility of hosts upon asymmetric *Wolbachia* infections, known as cytoplasmic incompatibility, CI^4^. These characteristics have heightened interest in using *Wolbachia* to control mosquitoes arboviruses^8,9^ and reduce pest populations via CI^10^. These manipulating experiments require a fine understanding of *Wolbachia* biology and diversity, and genomics can be a first step in this direction. Indeed, the long sought effectors of CI have been identified only recently after decades of research using comparative genomics^11,12^, but the major current limitation in *Wolbachia* genomics is that sequenced genomes are available from only a few hosts and strains (43 reference genomes at January 2018, Supplementary Data 3).

Although the main route of a *Wolbachia* transmission is from mother to offspring, horizontal transfer between host species (interspecific transmission) and loss of infection is common to the point that *Wolbachia* and host phylogenies do not agree^13,14^. The degree of Wolbachia intraspecific horizontal transfer (transmission between individuals of the same host species) is less documented but has been shown to play a key epidemiological role for example in cherry fruit flies^15^. Co-phylogenetic studies based on genomic data have found no clear evidence of intraspecific transmission in the fly *D. melanogaster*^16^ nor in the nematode *O. volvolus*^17^ and could not address this issue in *D. simulans* due to lack of phylogenetic signal^18^. Understanding whether *Wolbachia* generally transmits only vertically intraspecifically would require whole-genome analyses of more host populations as PCR based screening such as Multi Locus Sequence Typing (MLST) does not have sufficient discriminating power^19,20^.

*Wolbachia*, like most other endosymbionts, cannot be cultured in isolation. For this reason, and even when protocols for *Wolbachia* enrichment are used^21^, the host genome is routinely co-sequenced in the same experiment. This creates potential coverage and contamination issues as the host genome is typically two to three orders of magnitude larger than that of the symbiont. This however also presents an opportunity as shotgun sequences targeting animals may serendipitously contain traces of their symbionts which might then be extracted to assemble genomes. Recovery of *Wolbachia* genomic DNA present in *Drosophila* sequencing data^16,18,22^ has previously been demonstrated, including a recent effort to screen available sequencing projects using three *Wolbachia* genes as queries^23^.

To increase the number of available *Wolbachia* genomes, here we present a comprehensive and systematic screening of deposited Ecdysozoa sequencing projects. We use a novel combination of assembly-free^24^ and assembly-based tools (see Methods) to recover whole-genome information for more than 1000 newly metagenome-assembled genomes (MAGs). Although MAGs cannot reach the quality of isolate genome sequencing which is unavailable for intracellular parasites, this large catalog of *Wolbachia* MAGs allows us to infer robust phylogenies, identify new variants, build host population-level datasets, and ultimately clarify some open questions concerning *Wolbachia* evolution.

## Results

### Expanding *Wolbachia* genomics with 1,005 newly assembled genomes

We screened for the presence of *Wolbachia* sequences in more than 70TB of raw DNA sequencing data from 31,825 publicly available genome sequencing samples from more than 500 putative host species ranging from nematodes and insects to crustaceans, but also including various non-ecdysozoan species (Supplementary Data 4). Raw reads were mapped against a set of 43 decontaminated available *Wolbachia* draft genomes using a PanPhlAn^24^-based pipeline (see Methods).

**Fig. 1:**
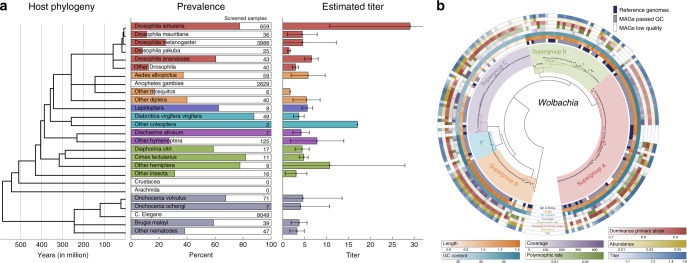
Prevalence, titer, and genome characteristics of *Wolbachia* in the analyzed samples. **a** Host related prevalence and estimated titer of more than 30,000 screened samples in relation to the host species phylogeny based on Rota-Stabelli et al.^5^. *Wolbachia* prevalence is based on the fraction of host samples in which *Wolbachia* strains could be detected at 1X or higher coverage using PanPhlAn. Statistics are reported only for host species with at least 7 samples. Infectious titers of *Wolbachia* cells per host cell are estimated from the fraction of reads and normalized by *Wolbachia* and host genome size taken from http://www.genomesize.com/. Error bars indicate the standard deviation over all titers of samples from the same host species. **b** PhyloPhlAn tree based on 1166 assembled genomes; reported for each genome are genome length, nucleotide composition in terms of G+C content, depth of coverage, fraction of nucleotide polymorphisms and dominance of the primary strain, relative read abundance, and estimated titer (normalized abundance). The large branches of *D. melanogaster* and *D. simulans* are excluded and duplicated sequences are not shown for clarity.

We found a total of 1793 samples (5.63%) positive for *Wolbachia*. This general low infection frequency is due to many samples being from model organisms including *Aedes aegypti, Caenorhabditis elegans* and *Anopheles gambiae* which are supposed to be *Wolbachia*-free or only very rarely infected with *Wolbachia*^25^. We verified the exact identity of all host species by reconstructing their 18S rRNA small subunit (see Supplementary Information and Supplementary Data 5). We found large differences in the level of infection frequency (prevalence) in the various hosts (Fig. 1a and Supplementary Data 4)) with values ranging from 100% in *Diachasma alloeum* to <5% in *Nasonia vitripennis*. Large differences exist even within the same genus. *Wolbachia* prevalence in *Drosophila simulans* and *D. ananassae* is twice of that in *D. melanogaster* and almost ten times higher than in other *Drosophila* species, confirming observations of similar large variations reported in other genera^26^. Despite consistency in the analytical pipeline used here across samples, these quantitative prevalence results should be taken with care because of likely sampling biases not traceable in sequence archives such as the presence of antibiotic-treated host strains, differences in DNA extraction protocols, and natural versus laboratory environments. The number of *Wolbachia* cells per host cell (normalized by genome size to approximate the actual titer) was also quite dissimilar with most estimates ranging from 3:1 to 10:1 (*Wolbachia* cells: host cells); exceptions are *D. simulans* (infected by wRi, 25:1) and the coleopteran *Callosobruchus chinensis* (infected by our newly assembled wCch 17:1).

We then metagenomically assembled 1166 genomes—called metagenome-assembled genomes (MAGs)—from our 1793 positive samples (see “Methods”), by selecting those characterized by high *Wolbachia* coverage. We verified that our *Wolbachia* MAGs did not contain host genome integrated fragments (see Supplementary Information), and excluded contaminations from other bacteria. By mapping all MAGs against nearly 100,000 bacteria reference genomes, we found only one instance of contamination. Using a genome-wide maximum-likelihood approach with PhyloPhlAn^27^ (Fig. 1b) we revealed a highly heterogeneous distribution of various genetic characteristics (genome length, nucleotide composition) and biological traits (normalized abundance: see Methods and Supplementary Fig. 2). In some cases the phylogenetic structure of *Wolbachia* could be linked with certain traits as in the case of supergroup C characterized by low GC content and reduced genome length, or supergroup D characterized by high abundance.

**Fig. 2:**
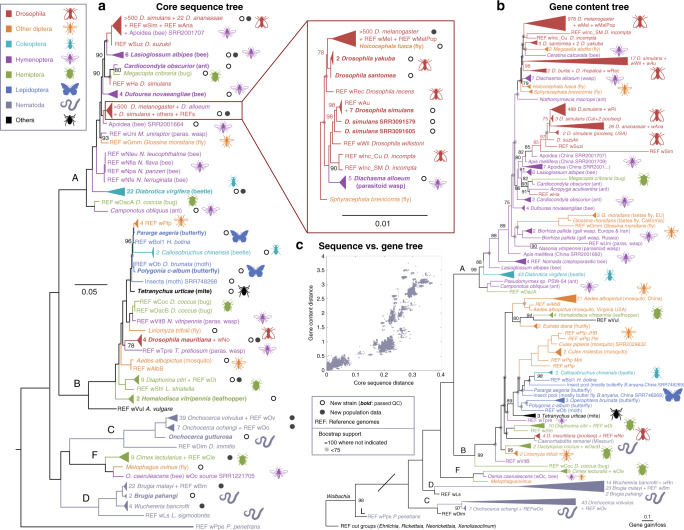
Whole-genome phylogenies of *Wolbachia*. **a** Maximum likelihood tree of 313 core genes alignment employing 339,972 nucleotide positions from 1161 newly assembled genomes (1005 genomes complied with our quality criteria plus 156 of core gene quality, see “Methods” section and Supplementary Data 2). Larger clades have been collapsed and their subtrees are represented by triangles. Bold host species indicate assembled genomes that fulfilled our quality requirements. The complete tree including support values is available in Newick format (Supplementary Data 1). **b** Functional potential tree based on presence and absence of 6376 gene families in 1793 *Wolbachia* positive samples as inferred by PanPhlAn. Gene families present in <5 *Wolbachia* positive samples were not considered. Both trees were inferred with RAxML using the GTR+G model of replacement and the BINGAMMA model, respectively, in both cases bootstrapping with 100 pseudo-replicates (for support see legend). **c** Scatterplot showing pairwise distances between all possible genome pairs in the core gene sequences (tree in **a**) versus identical pairs in the binary tree based on gene presence (**b**). Animal silhouettes have been created by MS.

We defined MAGs quality based on four main criteria (see “Methods” and Fig. 1b) as a strict control to retain a total of 1005 *Wolbachia* MAGs, which we have used to infer a whole-genome phylogeny based on the alignment of 316 core genes (Fig. 2a). Although MAGs are inherently less accurate than genomes obtained by isolate sequencing, the resulting maximum-likelihood tree is overall very robust with most nodes receiving full (100% bootstrap) support. We complemented this phylogeny with an analysis of the presence and absence of the 6376 gene families composing the pangenome of the 1793 Wolbachia-positive samples using PanPhlAn^24^ (Fig. 2b). The two trees show a similar topology as further indicated by comparison of branch lengths (Fig. 2c, ρ = 0.94, *p* < 1E–50, Spearman correlation). The position of supergroups C and D is however an exception. This is likely due to shared functional properties between F, A, and B, which may have promoted a Long Branch Attraction artifact^28^ of C and D with functionally distant outgroups. Overall, our analysis increased the diversity of available *Wolbachia* genomes by more than 60% reaching 1166 *Wolbachia* genome and increased by more than 50% the number of host species with an assembled *Wolbachia* genome (from 33 as of July 2018 to 55), providing an useful dataset for comparative genomic and phylogenetic studies (Supplementary Data 5).

### The catalog of reconstructed strains expands the *Wolbachia* sampled diversity

Our *Wolbachia* genome resource contains new MAGs of previously described data in four host species (*Drosophila melanogaster, D. simulans, D. ananassae, Onchocerca vulvus*)^16–18,29^ and crucially expands *Wolbachia* diversity with newly assembled genomes from both known and unknown hosts (white circles in Fig. 2a). For example, we enlarged the genomic diversity in D. *melanogaster* and *D. simulans* by adding new genomes to reach more than 500 quality controlled genomes each, including the *Wolbachia* genomes for *D. mauritiana*^30^, *D. yakuba* and *D. santomea*^31^. We similarly provide additional assembled genomes for various nematodes of medical relevance such as *Wuchereria bancrofti* and *Brugia pahangi*, and genomes in insect of economic importance such as the corn rootworm *Diabrotica virgifera* and the bedbug *Cimex lecturalis*. These collections of *Wolbachia* genomes from the same host (black circles in Fig. 2a) provided us with data for *Wolbachia* intra-host population studies, as addressed below. We also assembled many genomes for unrepresented insect orders Lepidoptera (e.g: butterflies *Polygonia c-album* and *Pararge aegeria)*, Hemiptera (e.g., the pest species *Homalodisca vitripennis* and *Dactylopius coccus*), and Hymenoptera (e.g: parasitoid wasps *Diachasma alloeum* and two bee species). We assembled the first *Wolbachia* genome from an arachnid host, the mite *Tetranychus urticae*. Overall, the majority of our newly assembled genomes are from insects and nematodes because hosts such as terrestrial crustaceans and arachnids are poorly represented in sequence archives. A detailed description of newly detected strains is given in Supplementary information.

### Evolutionary scenarios highlighted by the new *Wolbachia* MAGs

We found evidence of high host intraspecific *Wolbachia* genetic diversity. In some cases, this variability was geographically linked as for the gall wasp *Biorhiza pallida* and the ant *Cardiocondyla obscurior* (in purple in supergroup A, Fig. 2b), or in the medical relevant tiger mosquito *Aedes albopictus* and tsetse fly *Glossina morsitans* (orange in, respectively, supergroup A and B, Fig. 2b). We also found new strains in *D. simulans* which are robustly separated (high bootstrap support) from known variants resident in this host such as the *w*Au-like strains in the box of Fig. 2a. This finding points toward the peculiar role of *D. simulans* as a reservoir of *Wolbachia* diversity in nature with at least seven distinct *Wolbachia* genome types observed in this species.

In some cases we identified *Wolbachia* strains very closely related to well-known reference *Wolbachia* genomes that are however present in unexpected hosts. We found, for example, a genome from the robber fly *Holcocephala fusca* having a 99.52% core genetic identity with *w*Mel of *D. melanogaster*: Although 18S rRNA gene screening confirms this SRA as *Holcocephala*, we found some reads with high similarity to the cytochrome oxidase subunit I gene (COI) of hoverflies. We therefore cannot exclude that our reconstructed *Wolbachia* genome is not from the robber fly itself, but from a hoverfly prey. Because we can exclude contamination from a *Drosophila* prey (Supplementary Information), this strain likely indicates a recent horizontal transfer involving distant hosts and showcases the complexity of the *Wolbachia*-*Drosophila* symbiotic models. We further identified a dubious new strain in the nematode *Caenorhabditis remanei* with a 98.04% identity in gene content with *w*No of *D. mauritiana* (Fig. 2b). While we could verify the host based on 100% similarity with the annotated COI gene of *C. remanei*, we also found some reads covering a portion of *D. mauritiana* COI. Since no *Wolbachia* has ever been found in the *Caenorhabditis* genus, it is possible that this *Wolbachia* is the result of some kind of contamination. Indeed, the genome from this sample did not pass our quality control and was considered only for our gene-content tree (Fig. 2b and not Fig. 2a). These two cases highlight the difficulty in determining the exact source of some samples when reconstructing endosymbiont genomes using a metagenomic approach.

As expected from a symbiont that can transfer horizontally between host species, we did not observe a strict or meaningful phylogenetic clustering of hosts in our *Wolbachia* phylogenies. We however identify some peculiar patterns, for example a clustering of Lepidoptera and a concentration of Hymenoptera in group A (respectively blue and purple in Fig. 2). The latter have a clear paraphyletic distribution on the tree consistent with a scenario where Hymenoptera in general (and not only parasitoids^32^) may have played a key role in the differentiation of *Wolbachia* supergroup A.

### Lineage-specific acquisition of gene families in supergroups

**Fig. 3:**
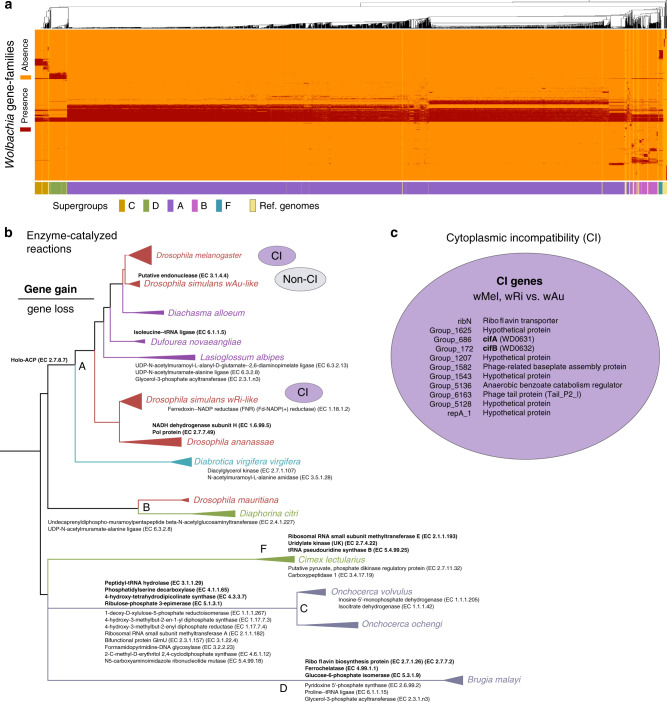
Functional analyses of *Wolbachia* pangenomes. **a** *Wolbachia* strain discrimination based on presence and absence of genes from the *Wolbachia* pangenome. Each column represents the identified gene content of a *Wolbachia* strain in one of the 1793 positive samples as detected by PanPhlAn. *Wolbachia* gene profiles are hierarchically clustered based on Jaccard distance. **b** Host specific functional gain and loss. Phylogenetic tree is based on *Wolbachia* genomes from hosts with at least 4 quality controlled assemblies. Fisher test was applied to identify host lineage-specific functional gain or loss of enzyme categories (EC numbers). **c** candidate genes significantly enriched in CI associated assemblies.

Our functional pangenome analysis (Fig. 3a) showcased large gene content divergence even within supergroups (in particular A). We performed a more stringent host-specific functional enrichment analysis by selecting 989 genomes from 14 different hosts with multiple assemblies. Investigating the functional enrichments and depletions (enzyme groups) on the phylogeny (Fig. 3b and Supplementary Fig. 1) we found that some functionalities have been independently lost in different host-specific lineages including for example the UDP-*N*-acetylmuramate-alanine ligase and the Glycerol-3-phosphate acyltransferase. Conversely, the Holo-acyl-carrier-protein synthase (EC 2.7.8.7) was instead acquired in the ancestral lineage of supergroup A. Overall, we observe more functional acquisitions and depletions in supergroups C, D, and F, than in supergroups A and B. This does not seem to be a bias related to accelerated mutation rate in C, D, and F, as their actual phylogenetic branch lengths (therefore number of mutations assuming a similar mutation rate) are similar to that of A and B (Fig. 2a). More likely, it reflects that most genomes in C, D, and F belong to highly adapted strains with degraded genomes.

To further identify putative genes responsible for CI, we compared strains known to cause CI with strains that are assumed not to possess this phenotype (Fig. 3c). Because of the relatively high gene variability between strains, the number of *Wolbachia* genomes considered here is crucial to pinpoint CI-specific genes. We compared 11 non-CI *w*Au-like assemblies with hundreds of CI related assemblies from *w*Ri-like and *w*Mel, using nematode lineages (*B. malayi* and *O. ochengi*) to correct for false CI gene loss (see Methods). For this analysis we selected only very similar genomes and made the assumption that genomes that are very similar to the ones causing (or not causing) CI also cause (or not cause) CI: in the absence of phenotypic information from a large panel of *Wolbachia*, our assumption was necessary to perform a pangenome analysis which is statistically robust against random sampling. We found that among the 11 candidate genes significantly enriched in CI inducing genomes (Fisher’s exact test, *p* < 10^−05^, Bonferroni corrected, Supplementary Data 6), five of them, including *cifA* and *cifB* were previously identified by LePage et al.^11^. Our pangenome analysis reveals six additional genes with functional annotations related to, among others, riboflavin, benzoate, and a bacteriophage. While significantly enriched phage genes might not be surprising, as the CI loci are located in a prophage region, other candidates may play a role in CI biology and should be investigated further experimentally.

### Correspondence of co-phylogenies and intraspecific horizontal transfers

**Fig. 4:**
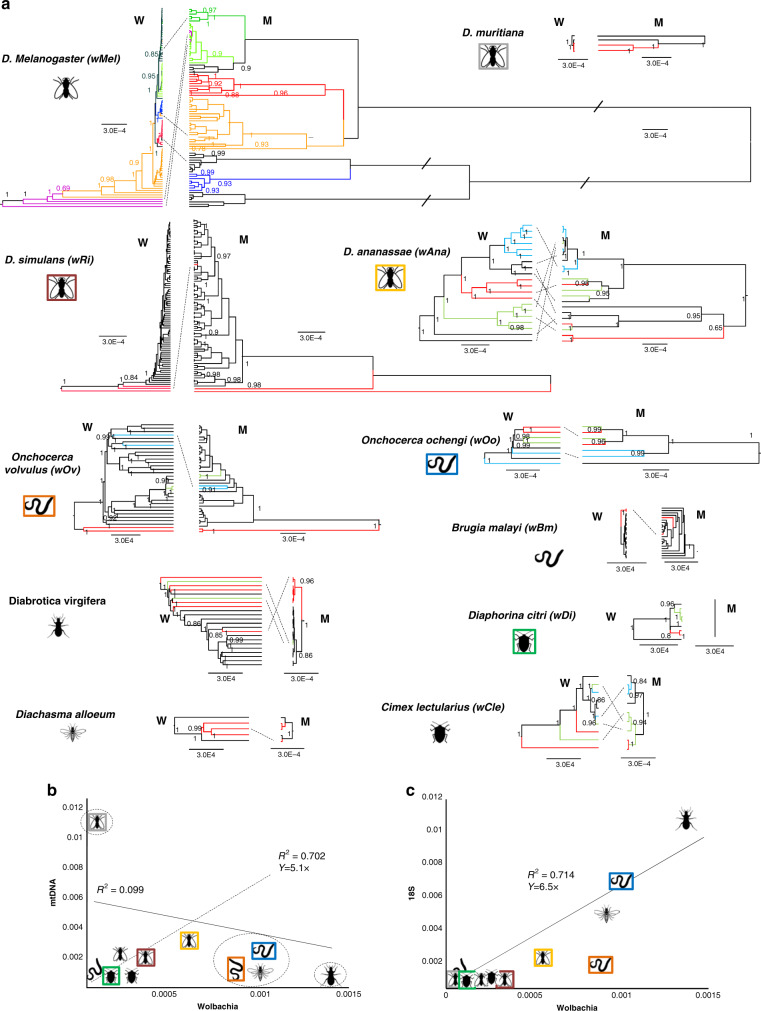
Co-phylogenies of *Wolbachia*, mitochondria, and 18S in eleven host populations. **a** Bayesian consensus trees of *Wolbachia* (W) and corresponding mitochondrial host (M) genomes from eleven species. All trees have been rescaled to the same rate (0.0003 mut/site) and colors were added to aid comparisons. A bar on branches indicates that the branch has been halved in length in order to fit the figure. Nodes below posterior probability 0.85 are considered poorly supported, and they are not considered to report a phylogenetic incongruence (dotted lines). **b** Average genetic distances (mutations per site) of each of the *Wolbachia* populations against the average genetic distance of the corresponding host’s mitochondrial genome. The dotted line is the regression in the absence of circled populations. **c** Average genetic distances of *Wolbachia* populations against the average genetic distance of the corresponding host’s ribosomal 18S. Full detailed versions of the trees, together with all NCBI-SRA names and BEAST xml files and genetic distances can be found in Supplementary Information and Supplementary Data 1.

Our extended genomes catalog allowed us to explore *Wolbachia* evolutionary dynamics within host populations, in particular, to assess the degree of intraspecific horizontal transmission. We used a reference-based mapping as in previous population studies^16,18,32^ to obtain longer *Wolbachia* genome alignments and assembled 1,149 mitochondrial genomes of the corresponding hosts to evaluate their co-phylogeny in eleven species (Fig. 4a).

Our co-phylogenies indicate a good correspondence between intraspecific host relationships and their corresponding *Wolbachia*: this is consistent with a prevalently vertical rather than horizontal inheritance as previously shown in *D. melanogaster*^16^. We found however numerous exceptions. All hosts species, except for the poorly sampled *D. mauritiana*, are characterized by one or more cases of phylogenetic incongruence which corresponds to likely cases of intraspecific horizontal *Wolbachia* transmission (dotted lines in Fig. 4a; the actual cases of horizontal transfer may be more than those apparent from our analyses because we considered only highly supported incongruences). For *D. melanogaster* and *D. ananassae* we observed some instances of horizontal transmission, in conflict with previous studies using less data^16,29,32^. We further found hints of peculiar inheritance mechanisms. Exceptional is the case of the bug *D. citri* which is characterized by clonal (identical) mitochondria, but whose *Wolbachia* population is well structured. This may reflect recent host infections from a pool of genetically diverse *Wolbachia* or a case of recent intraspecific transmission^15^. Of note is also the case of *D. simulans* whose *Wolbachia* are almost indistinguishable, while the host has some mitochondrial structure compatible with the recent rapid spread of *w*Ri within this host^33^. To check for possible contaminants that may have misled our co-phylogenies, we further explored possible host-derived sequences using the procedure of Chung et al.^34^ (see Methods) and found on average about 0.1% positions of potential sequence variant contamination across all assemblies. We detected various instances of increased sequence variations at 1–6% length, but only eight assemblies with increased coverage regions at 1–3% length mostly in nematode hosts. (Supplementary Data 5). Because Wolbachia-host lateral gene transfers (LGTs) shall generate a significant increase in coverage variation, we conclude that only eight of our MAGs are potentially affected by misleading signal related to LGT, None of these MAGs are involved in any of the putative within host transfers (dotted line of Fig. 4a) or exhibit any peculiar rate of evolution in Fig. 2a. However, because we found a certain level of genetic heterogeneity, we cannot completely exclude confounding factors from multiple Wolbachia infections in some of the MAGs. For *D. ananassae* we further manually excluded bias from *w*AnaINTs (*Wolbachia* genomes integrated in host genomes) data accidentally included in our assemblies, but could not find fragments attributable to integrated *Wolbachia* (see Supplementary Information); furthermore, as integrated genomes evolve neutrally, or almost neutrally^29^, they cannot produce a phylogenetic signal incompatible with that of the host genome. Because of its peculiar integrating genome biology, we nonetheless advocate caution in the interpretation of our results for *w*Ana.

### Rates of *Wolbachia* evolution correlates with nuclear, but not with mitochondrial genome of hosts

An intriguing outcome of our co-phylogenies is the striking heterogeneity of the evolutionary rate of *Wolbachia* compared to that of the host’s mitochondrial DNA (mtDNA). While we could reproduce previous finding that *Wolbachia* evolves about one order of magnitude (circa 10 times) slower than mitochondria in *D. melanogaster*^16^, we could observe a similar pattern only in *D. mauritiana*, and to a lesser extent in *D. simulans* and *D. ananassae*. In all other hosts we observe different patterns: *Wolbachia* rates are approximately the same as mitochondria in *Onchocerca* nematodes, and two/three times faster than mitochondria in the wasp *D. alloeum*, the beetle *D. virgifera* and the bedbug *C. lecturalis*. These discrepancies seem to correlate with host biology traits such as generation time and population size: while *Drosophila*, for example, is characterized by many generations per year and large effective population size^35^, most other species are characterized by less generation per year and smaller population size^36,37^.

Our co-phylogenies reveal major desynchronization between the *Wolbachia* and the mtDNA clocks. We did not infer co-phylogenies using host nuclear data because, while mtDNA and *Wolbachia* are uniparentally inherited, nuclear genes follow a Mendelian inheritance and their coalescence impedes building a genealogical (tree-like) structure^38^. We have however estimated average genetic divergence for a typical universal animal nuclear marker, the 18S rRNA gene^5^ and compared the average genetic distances of each of the 11 *Wolbachia* populations with that of the mtDNA and 18S of the corresponding hosts. As anticipated by the different relative rates of the co-phylogenies (Fig. 4a), there is no correlation between *Wolbachia* and mtDNA divergences (Fig. 4b). Instead, we found a significant correlation between *Wolbachia* and 18S rRNA gene evolution (Fig. 4b). This indicates that *Wolbachia* is indeed following the molecular clock of the hosts, but only for what concerns its nuclear genome, not the mitochondrial one. By contrast, the mtDNA seems to be characterized by a rate that departs from both *Wolbachia* and, apart from a few cases, the nuclear data. One possible explanation is that the mitochondrial genomes of 4-5 species are characterized by an unusual within-species genetic homogeneity possibly due to specific inheritance genetics or a constrained selective regime. Indeed, when we exclude these species (circled in Fig. 4b), we recover a significant correlation, and notably with a similar slope of the *Wolbachia*−18S correlation (dotted line in Fig. 4b). This finding deserves future testing in particular by analyzing large chunks of the host nuclear genome and by expanding these comparisons to more populations. Our data is however clear in showing that the pattern and rate of *Wolbachia* evolution may be very dissimilar to that of their mtDNA host, pointing at a variably and species-specific *Wolbachia*-mtDNA evolution, in which the *Drosophila-Wolbachia* dynamics^16^ seems to be the exception rather than the norm.

## Discussion

We have investigated the global *Wolbachia* genomic diversity by de novo assembly and analysis of more than a thousand genomes. With 1.793 positive samples and 1005 assembled genomes, 40 of which are from new host species, we show that our metagenomics approach is effective in retrieving large amounts of endosymbiont genome data. Overall, we could show that our approach provides a highly robust enhanced picture of both deep time and shallow time *Wolbachia* evolution, which may be used as an actual reference for future studies. We advocate that the metagenomic approach and pipeline we have used here should be extended to other less known unculturable endosymbionts such as *Spiroplasma* or *Cardinium*^40^.

Our data allowed us to explore more in depth *Wolbachia* diversity. We found new variants related to model *Wolbachia* strains such as *w*Mel and *w*Ri as well as new genomes from medically and socio-economically relevant organisms including mosquitoes, pests of agriculture, and nematodes. Our phylogenies provide a robust reference for future *Wolbachia* studies and the branching position of parasites and parasitoids in our phylogenies invigorate the idea that they may have played a key role in the horizontal transfer of this bacteria.

Probably the most exciting outcome of our study is the between-hosts heterogeneity of *Wolbachia* evolution rate in comparison to mitochondrial rate: in some hosts *Wolbachia* evolves 10 times slower than mitochondria, in other hosts at the same rate or even faster than mitochondria. We hypothesize that the reason behind this large rate variability is not due to *Wolbachia*, but rather due to peculiar mitochondrial genetics in some hosts: this is because rates of *Wolbachia* evolution correlates with nuclear, but not with mitochondrial genome of hosts. This has important outcomes for comparative studies: mutation rate inferred from the mitochondrial rate of model organisms such as *D. melanogaster*^16^ should be used with care when calibrating *Wolbachia* evolution in other hosts^39^.

## Methods

### Retrieval of existing *Wolbachia* reference genomes

The reference set of *Wolbachia* genomes was compiled with the sequenced strains publicly available as of January 2017. We retrieved 41 of the 43 public available *Wolbachia* reference genomes from RefSeq^41^ and GenBank^42^, whereas the genomes of strains wDi and wLs which were downloaded from http://nematodes.org/genomes/index_filaria.html^43^ because these two genomes were not yet deposited in NCBI. This set includes 19 genomes of *Wolbachia* supergroup A, 15 of supergroup B, four of supergroup C, three of supergroup D, one of supergroup F, and one of supergroup L. The NCBI accession numbers for all *Wolbachia* genome sequences used for phylogenetic analysis are given in Supplementary Data 3. To avoid potential biases due to different genome annotation procedures, all genomes were re-annotated using PROKKA^44^ (version 1.11).

### Retrieval of raw-sequencing data from host (re)sequencing projects

Public shotgun sequencing samples were retrieved from the NCBI Sequence Read Archive (SRA) database by selecting all available sequencing projects that targeted organisms in taxonomic families that can host *Wolbachia* endosymbionts. The database search was based on 181 arthropod and nematode host-related keywords (common names such as insect(s), worm(s), mite(s), plus various taxonomic units ranging from Phyla to Genera; see Supplementary Information) and resulted in a total number of 31,825 samples (72.3TB of raw-sequencing data, Supplementary Data 4)).

### Identification of *Wolbachia*-positive samples

We first screened for the presence of *Wolbachia* in the samples to identify the samples that were analyzed with the genome and pangenome reconstruction pipelines (see below). In this screening step, the sequences of all samples were mapped against available *Wolbachia* reference genomes using the Bowtie2^45^ aligner (version 2.1.0). Breadth of coverage for each reference genome was calculated using Samtools^46^. For follow-up analysis, we selected the 1,793 samples that show a breadth of coverage larger than 50% of at least one reference genome (at 1X depth of coverage level). All these samples were used for the pangenome analysis (PanPhlAn^24^). The 1220 samples with an average depth of coverage for a *Wolbachia* genome larger than 4X were selected for the assembly-based approach.

### *Wolbachia* prevalence and estimated titers

For host species with multiple screened samples, we identified the *Wolbachia* prevalences as the proportion of infected subjects found in a host species population (Fig. 1). Prevalences are estimated for strain detection at a low >1X coverage level using PanPhlAn and for higher >10X coverage as required for assembly. Supplementary Data 4) includes the prevalences for all host species in which we could detect at least one *Wolbachia* strain. Non-infected (zero prevalence) host species are only included for large screens of 50 or more samples from the same host. The reported prevalence levels might be affected by the sometimes limited metadata quality of the NCBI repository and our sampling strategy. Host species tree topology and divergences (Fig. 1) are obtained following Rota Stabelli et al.^5^ and Ometto et al.^35^. Infectious titers are estimated for each assembled *Wolbachia* genome based on the abundance ratio between *Wolbachia* and host sequences, and normalized by host and *Wolbachia* genome lengths (Supplementary Data 3).

### Assembly-free Wolbachia pangenome analysis using PanPhlAn

We applied PanPhlAn^24^ to characterize the strain-specific gene repertoire of the *Wolbachia* genomes in the positive samples. In its first step, PanPhlAn estimates the abundances of all genes in the *Wolbachia* pangenome. Then, it uses a co-abundance criterion to define the set of genes specific to the strain present in a sample. The *Wolbachia* pangenome, consisting of all genes known to occur in a *Wolbachia* strain, was generated by merging genes from all 43 *Wolbachia* reference genomes. Homologous genes are clustered into gene families with a nucleotide identity threshold of 80% using Roary^47^, version 3.5.9, which resulted into a set of 10,725 distinct pangenes. For all 1793 pre-selected samples, pangene profiles were generated using a minimum coverage threshold of 1X (“–min_coverage 1” option) which allow profiling also those strains that cannot be assembled because of low coverage.

### Wolbachia (metagenomic) assembly and annotation from host sequencing data

We applied metagenomic assembly to each of the 1220 *Wolbachia*-positive samples having a coverage of at least 4X. 1005 MAGs passed our quality control criteria as defined below. Since some MAGs of lower quality are derived from exceptional hosts, for reconstructing the phylogenetic core tree, we used all 1161 MAGs that contain at least 33% core genes (Supplementary Data 5). 54 MAGs are rejected as not fulfilling any quality criteria. Assembly was performed with MegaHit^48^ version 1.0.5. Contigs shorter than 1000 nt were removed. Because of the availability of a large set of *Wolbachia* reference genomes, we used a taxonomy-based binning approach to group contigs into draft genomes. This pipeline starts by mapping all the assembled contigs against all *Wolbachia* reference genomes using BLASTn^49^ with the “task blastn” option that uses a shorter word-size of 11 bp, thereby allowing to align more distantly related sequences. A contig was included into a *Wolbachia* draft genome if it mapped against any *Wolbachia* reference genome with >75% identity over a fraction of the length of the contig higher than a threshold T. The value of T was chosen so that short contigs were required to overlap almost entirely with the reference while longer contigs were only required to align at a shorter fraction with a reference genome in order to allow the discovery of new *Wolbachia* accessory sequences. We thus used a logarithmically scaled value such than short 1000 bp contigs were required to be aligned over the full length of the contig, 2,000 bp contigs over 65% of the length, and contigs longer than 10,000 bp over 20% of the length. Precisely, for a contig with a total length *L*_c_ and aligned length *L*_a_, we require: *L*_a_ / log_10_(*L*_c_/100)/1000 > 1. The final contig sets of assembled *Wolbachia* draft genomes were annotated using PROKKA. Information about the draft genomes, including the NCBI source sample SRA number and host species, are given in Supplementary Data 5.

### Quality control of the assembled *Wolbachia* genomes

To select quality controlled genomes, we filtered out assembled *Wolbachia* genomes that (i) showed a genome coverage lower than 20X, (ii) contained less than 50% core genes (see below), (iii) had a total length shorter or longer than the range 750,000–1,700,000 bp (set based on the lengths of *Wolbachia* reference genomes), and finally (iv) showed evidence of chimeric assemblies due to the presence in the corresponding sample of more than one *Wolbachia* strain. For detecting *Wolbachia* coinfections, point (iv), we generalized the approach we described for StrainPhlAn^50^ to identify polymorphisms in the reads used to build the assemblies. The number of polymorphic sites in the assembled genomes gives a direct indication of the presence of one or more *Wolbachia* genomes in the same sample. A high polymorphic rate can indicate potential incorporation of multiple populations into a single MAG or incorporation of host sequence of lateral gene transfer (LGT) events. In detail, the procedure starts by mapping with Bowtie2^45^ all the reads of a sample against the *Wolbachia* genome assembled from the same sample. The resulting alignment was processed using Samtools^46^ to detect polymorphisms in the reads mapping against each position of the assembly. Based on a probability mass function of a binomial distribution, we label as polymorphic those positions for which we reject the null hypothesis (alpha 0.05) that only one base is present. We consider an error rate of 0.01 (i.e. 1%) for Illumina sequencing reads and we only consider read bases with a quality higher than 30. Once all positions of the assembly are assessed, we consider the fraction of the assembly length with significant polymorphisms. When this fraction is greater than 0.05 (more than 5% polymorphic sites) we assume that multiple *Wolbachia* is present in the same sample. Assembled genomes are thus considered as of reasonable quality when the polymorphic rate is lower than 0.01 or between 0.01 and 0.05 but showing a dominance of the primary strain in the polymorphic regions larger than 80%. Polymorphic rates are shown in Supplementary Fig. 2. Additionally, we screened our assemblies for low-confidence regions defined by unexpected higher coverage and higher sequence variation compared to average, following the procedure of Chung et al.^34^. We extracted all ≥50 bp regions with a sequencing depth of ≥4 median coverage and all ≥50 bp regions with ≥4 average sequence variation (positions with secondary base ≥5% coverage). To control for potential sequence contamination, we mapped all our passed QC assemblies against nearly 100,000 bacteria reference genomes from NCBI GenBank. Using a BLAST threshold of >97% identity over a minimum alignment length of 10,000 nucleotides, we identified only one *Drosophila melanogaster* related *Wolbachia* strain (SRR1183652) that includes sequences that are similar to *Acetobacter* related species. Additionally, we could confirm the quality of our assemblies by re-assembling *Wolbachia* reference genomes from available source samples. Based on a core sequence length of more than 300,000 nt, we found 99.99% genetic identity between our assemblies (SRR183690, SRR1508956) and the original reference genomes of *w*Di and *w*Rec, and even 100% core identity between our assembly (ERR188908) and the reference strain *w*Suzi. To confirm NCBI metadata about the host and to clarify samples tagged as “Apoidea“ and “Insecta”, we verified the host species by reconstructing the 18S ribosomal RNA gene sequence using the tool RiboTagger^51^. Based on all these criteria, we selected 1005 assemblies that fulfilled our quality requirements. The assembly quality measures for the genomes are given in Supplementary Data 5.

### Pangenome analysis and core-genome identification

In a first step, we generated the *Wolbachia* pangenome across all assemblies using Roary^47^ version 3.5.9 with a BLAST identity cutoff of 80% and paralog splitting disabled. We then identified the *Wolbachia* core genes by applying an approach that balances the different numbers of assembled genomes in the *Wolbachia* supergroups. To get an unbiased set of core genes, we selected a set of five representative genomes from each supergroup as follows: We clustered all genomes of each supergroup using *k*-medoids clustering (*k* = 5, Jaccard distance) applied on Roary gene presence/absence profiles. The central genomes of each of the five clusters are selected as representative genomes of a supergroup, which in total are 25 genomes covering the variation of *Wolbachia* supergroups A, B, C, D, and F. We generated the pangenome of the 25 representative genomes using Roary with a BLAST identity cutoff of 80% and paralog splitting disabled. The final core gene set was created by selecting gene-families that are present in at least one of the five representative genomes in each *Wolbachia* supergroups. In total, we could identify 316 *Wolbachia* core genes from the representative genomes present in all considered supergroups. We then used our *Wolbachia* core gene set to compare all 1200 assembled genomes by extracting the individual core gene sequence for each genome. For each core gene-family, we mapped the gene sequences of the 25 representative genomes against the gene sequences of each genome using BLASTn (task type blastn and word-size = 7 bp). A matched sequence was selected as core gene sequence based on the following thresholds: E-value < 1e-10, percent identity >66%, and the alignment length was required to be not shorter than 50% of the average core gene sequence and not longer than 125%. To identify single-copy genes, we excluded three core genes that show multiple hits in more than ten percent of our assembled genomes. For all remaining 313 core genes, we selected only gene sequences that are present as a single hit.

### Phylogenetic analysis

Based on the identified *Wolbachia* core gene set, we constructed a comprehensive phylogenetic tree covering five different *Wolbachia* supergroups by using all assembled genomes that contain at least 33% core genes. We aligned each core gene family using Mafft^52^ version 7.215 with globalpair G-INS-i strategy and we then concatenated the alignments. We constructed the maximum-likelihood tree (Fig. 2a) under the GTR plus GAMMA model using RAxML^53^ version 8.2.9. Node support was estimated with 100 bootstrap replicates.

### Functional analysis

To compare the gene content of our assembled *Wolbachia* strains, we characterized the core and accessory genome of the overall *Wolbachia* population and compared 14 host-specific lineages. Comparison is based on Roary gene clustering as described above. Fisher exact test (*p* < 0.05) was used to identify lineage-specific gene gain and loss (Fig. 3, Supplementary Data 6). Each host-specific lineage was separately compared to all closely related lineages. Gain or loss of genes are only reported if coincident across all comparisons. To identify CI genes, we compared non-CI *w*Au-like genomes independently with both CI lineages *w*Ri-like and *w*Mel, excluding 79 genomes that show an identical core sequence to other genomes. Only genes that are significantly enriched in both comparisons are reported as CI candidate genes. Additional comparisons of both CI lineages with nematode lineages (*B. malayi*, *O. ochengi*) are performed to reject incorrect CI gene loss due to genes enriched in the non-CI *w*Au-like lineage.

### Co-phylogenies including MUMmer alignments and mitogenomes

Host-specific multiple genome alignments of *Wolbachia* sequences were constructed for the 11 species in Fig. 4 using a modified StrainEst^54^ pipeline. Briefly, for each host species a reference genome of *Wolbachia* was chosen and all other genomes were aligned against the reference using the nucmer command from the MUMmer suite^55^. Ambiguous mappings (i.e., regions that can be mapped against more than one region) in alignments were discarded. In order to reduce the size of the datasets in the case of strains infecting *D. melanogaster* and of wRi strains infecting *D. simulans*, genomes were filtered by discarding those of total length <1.1 Mbp. In addition, since aligning a large number of almost identical draft genomes could severely reduce the size of the core genome due to the stochastic distribution of missing sequences, for these two datasets an all-vs-all distance matrix was computed using Mash^56^, and sequences were clustered using complete linkage hierarchical clustering with a threshold of 0.005, keeping only one representative sequence for each cluster for downstream analysis. From the nucmer pairwise sequence alignments to the reference a host-specific core genome alignment was built.

We reconstructed the mitochondrial DNA (mtDNA) of eleven hosts by using a reference-based read mapping. Short reads were aligned with the corresponding host mtDNA reference using Bowtie2. A consensus sequence was built from all covered reads using samtools mpileup and a majority rule to merge the bases across reads. Host-specific mtDNA sequences were subsequently aligned using Mafft with globalpair G-INS-i strategy.

We inferred phylogenies of the eleven sets of *Wolbachia* and corresponding mitochondrial genomes using BEAST^57^ version 1.10 and employing for both types of datasets a GTR + G replacement model, a coalescent tree prior, strict clock, and no calibrations priors in order to have phylogenies scaled only on mutations per site. We repeated the analyses using RAxML employing a GTR plus GAMMA replacement model (as detailed above) to further test phylogenies. For computational reasons, we inferred BEAST analyses of *Wolbachia* from *D. melanogaster* and *D. simulans* after exclusion of invariable sites: for consistencies, tree topologies have been rescaled according to RAxML branch lengths inferred using whole alignment.

### Reporting summary

Further information on research design is available in the Nature Research Reporting Summary linked to this article.

## Supplementary information

Supplementary Information

Peer Review File

Reporting Summary

Description of Additional Supplementary Files

Supplementary Data 1

Supplementary Data 2

Supplementary Data 3

Supplementary Data 4

Supplementary Data 5

Supplementary Data 6

Supplementary Data 7

## Data Availability

All assembled *Wolbachia* genomes described in Fig. 2 have been deposited at the European Nucleotide Archive (ENA) under project PRJEB35167. All trees and corresponding xml files are in Supplementary Data 1–2. Reference genomes, download statistics, individually assembled genome with accession numbers and statistics, functional data, and genetic distances are provided in Supplementary Data 3–7).

## Code availability

All relevant codes used in this work are available, upon request, from the corresponding authors (N.S. or O. R-S).
