# Supplementary material for: Large scale genome reconstructions illuminate Wolbachia evolution
Source: Nat Commun. 2020 Oct 16;11:5235. doi: 10.1038/s41467-020-19016-0 (PMC7568565; doi:10.1038/s41467-020-19016-0)

Large scale analysis of over a thousand *Wolbachia* genomes sheds new light on its evolution.

Matthias Scholz[1,2], Davide Albanese[1], Kieran Tuohy[1], Claudio Donati[1], Nicola Segata[2], Omar Rota-Stabelli[1]

[1] Research and Innovation Centre, Fondazione Edmund Mach (FEM), San Michele all'Adige, Italy

[2] Department CIBIO, University of Trento, Trento, Italy

Correspondence and requests for materials should be addressed to NS (email: nicola.segata@unimi.it) and O R-S (email:omar.rota@fmach.it).

Supplementary information

Supplementary Notes

1. Detailed description of novel strains and affinities in the phylogenetic trees of Fig. 2

General phylogenetic considerations

Although they support very similar topologies, the presence-absence tree (Fig 2b) is generally less supported than the alignment tree (Fig. 2a). This may be caused, at least partially, by some homoplasies (independently shared genes) in the presence/absence tree and by the sequence alignment being two orders of magnitude larger, therefore more robust to bootstrapping.

All genomes assembled in our analyses and depicted in Fig 2 belong to group A, B, C, D, F; none are from other less known groups such as E, M, or L. The most likely explanation is that the latter super-groups lack a high quality reference genomes: our pipeline uses a stringent annotation criteria to obtain high quality assemblies, which does not allow to identify *Wolbachia* genomes very

distant from known high quality reference ones. We foresee that our method will further increase Wolbachia sample diversity as soon as new high quality genomes will be available from less represented supergroups.

Supergroup specific phylogenetic considerations

Supergroup A

Even though the large inner branch of **supergroup A** is mainly composed of *Wolbachia* present in Diptera (flies) and Hymenoptera (bees, wasps, ants), we also found a related strain in Hemiptera *Megacopta cribraria* (bug) centrally located in the same branch, and closer to the root located strains from the Coleoptera *Diabrotica virgifera virgifera* (beetle) and the Hymenoptera *Camponotus obliquus* (ant) closely related to the reference wDacA (Hemiptera). We show enlarged diversity of genomes in various *Drosophila* species, including >500 *D. melanogaster*, >500 *D. simulans*, and 22 *D. ananassae*. We also assembled two new genomes from *D. yakuba* and one from *D. santomea*, located at the root of the *D. melanogaster* branch. Particularly interesting is an additional new genome from the fly *Holcocephala fusca* located close to the root of the *D. melanogaster* cluster. We also increased the diversity of *Diachasma alloeum* (wasp) by 7 genomes and assembled new *Wolbachia* genomes from the flies *Megaselia abdita* and *Sphyracephala brevicornis*.

Supergroup B

We expanded **supergroup B** by providing the first representative *Wolbachia* genomes of several hosts. The deepest branch in the supergroup B contains *Wolbachia* strains from Lepidoptera (butterflies, moths) which were not present in other supergroups. We found a new strain in *Polygonia c-album* (butterfly) closely related to the reference wOb from *Operophtera brumata* (moth). And we assembled a new strain from *Pararge aegeria* (butterfly) which is closely related to the references wPip (mosquito) and wBol1 (butterfly). We also found a very distinct strain in *Callosobruchus chinensis* (beetle) closely related to a new strain from an unidentified insect. Finally, the position closely located to the root of this Lepidoptera dominated branch is occupied by a new strain from *Tetranychus urticae* (mite). The central part of supergroup B is mainly composed of Hemiptera. We enlarged the diversity of *Diaphorina citri* (bug) by 9 genomes and we found a new strain in *Homalodisca vitripennis* (leafhopper).

Supergroups C, D, F

For the insect related **supergroup F** embedded in the nematode branch, we increased the diversity of *Cimex Lectularius* (bed bug) and, we assembled a new *Wolbachia* genome from *Melophagus ovinus* (fly) which shows to be closely related to *Wolbachia* wOc from *Osmia caerulescens* (bee). In the nematode **supergroup C**, we found a novel *Wolbachia* strain from

Onchocerca gutturosa which is placed at the root of the largely expanded Onchocerca cluster consisting of 39 *Onchocerca volvulus* and 7 *Onchocerca ochengi* genomes. In **supergroup D**, we increased the diversity by 22 *Wolbachia* genomes from *Brugia malayi* and 4 from *Wuchereria bancrofti*, and we provide 2 new *Wolbachia* genomes from *Brugia pahangi* that form a distinct branch closely related to *B. malayi*.

2. Integration analyses in *Callosobruchus chinensis* and *Drosophila ananassae*.

For *Callosobruchus chinensis* and *Drosophila ananassae*, we manually inspected our core-genome data (the one used for our main tree of figure 2). According to Choi et al. (2015, GBE) integrated *Wolbachia* are characterised by an excess of non synonymous mutations as well as stop codons and frameshifts because of relaxed selection on the integrated *Wolbachia* compared to non-integrated one. We first realigned the core genome for these two hosts in order to recover the codon frame of genes (the core genome is mainly composed of conserved Wolbachia coding genes), we then look for intergenic stop codons, frameshifts, as well as for region poorly aligned. We did not find any internal stop codon nor disruptive insertions/deletions, except for few poorly aligned fragments, which we have been blasted: they all look genuinely as *Wolbachia*:

- In one of the two *Callosobruchus Wolbachia* (SRR949786), we found a poorly aligned sequence at position 22673 of the core genome alignment (position 4588 of Callosobruchus alignment) 1638 nucleotide long. This regions however does blast with 99.5% identity (and zero gaps) against well annotated *Wolbachia* genomes such as wPip and wAlbB.
- In *D. ananassae* in SRS2127163 we found a 1757 nt fragment at position 19245 of the core genome) which however blast with 100% similarity to *Drosophila ananassae* strain W2.1 chromosome (wAna, cp042094.1) and with wRi (CP001391.1).
- In *D. ananassae* SRS2127151-2127152-2127153 we found a 489 nt fragment at position 6132 which blast 100% with *Drosophila ananassae* strain W2.1 chromosome (wAna, cp042094.1) and wRi (CP001391.1).
- In *D. ananassae* SRS2126857-2126916-21235641-2127154 at position 36570 a fragment of 783 nt blasting 100% with *Drosophila ananassae* strain W2.1 chromosome (wAna, cp042094.1) and wRi (CP001391.1).
- In *D. ananassae* SRS2126857-2126916-21235641-2127154-2135644-2135642 at position 181210 of 993 nt blasting 100% with *Drosophila ananassae* strain W2.1 chromosome (wAna, cp042094.1) and wMel_ZH26 (CP042445.1)

The fact that all these fragments blast with perfect or nearly perfect similarity with well-curated reference genomes such as wRi, wMel and wPip, and that we did not found any present insertion/deletion when compared to them reassured us that they are genuinely *Wolbachia* fragments and not host integrated fragments.

Furthermore, we also inspected our MUMmer alignment of *D. ananassae Wolbachia*, and did not find any evidence of wAnaINT: because according to Choi et al. 2015 (table 2 and table 3 therein) wAnaINT accumulate 20 times more mutations than wAnaINF, we would expect an excess of

mutations (therefore long branches in a phylogeny) in samples contaminated by wAnaINT fragments: the branch length of all samples is instead homogenous (similar to each other) very similar according to a RAxML analysis of the dataset.

3. Host verification

We verified the host species by reconstructing the 18S ribosomal RNA gene sequence using the tool RiboTagger. In all cases of successfully 18S reconstruction, we could not find any clear mislabelling of the host species. The host in the majority of our samples could be directly confirmed at the species level. Remaining samples could not be distinguished at species levels, but were confirmed at higher taxonomic levels, such as genus, and subgroup because annotated 18S are missing from the reference database. In few samples we could not extract the rRNA sequence, as the host sequences might have been removed and only the metagenome was made public (*D. recens*, the source data for wRec is one of these cases). For two species, *Diabortica virgifera* and *Drosophila ananassae*, RiboTagger could not confirm the taxonomic assignment of their SRA sample, respectively finding likely contaminant fungi or the fruit fly *S. lebanonensis*. This may be due to an issue in the RiboTagger pipeline or available references. We manually inspected COI and could confirm that the best blast hits of those samples were reciprocally with *D.virgifera* and *D.ananassae* (and no hit were found with *S. lebanonensis* in these samples).

We performed extra validation for two peculiar cases: *Holcocephala fusca* and C*aenorhabditis remanei.* At the genus level, we clearly could confirm the host *Holcocephala fusca* (SRR1738186) to be similar to the 18S reference of *Holcocephala abdominalis* 18S reference (the 18S of *H. fusca* is not yet deposited in GenBank). We further check for signs of contamination by blasting the SRR1738186 using COI of *Drosophila melanogaster* as query and found various reads with 95% similarity: blasting of these reads against the nucleotide collection returned as best hits other dipterans of the Syrphidae family (hoverflies such as *Sphaerophoria* sp., *Eristalis* sp, *Dasyhlea* sp). It is therefore possible that the wMel-like *Wolbachia* is from hoverfly prey (but not from a *Drosophila ad the COI found in* SRR1738186 does not have highest hits to them) of the robber fly. For *Caenorhabditis remanei* (SRR275642), we could not reconstruct any 18S sequence using RiboTagger due to low sequencing depth, but this sample is considered to be of lower quality, present only in the gene-content tree (Fig. 2b), and not part of our genome set in the core sequence tree (Fig. 2a). We manually blasted SRR275642 using *Caenorhabditis remanei* Cytochrome Oxidase subunit I (COI) and found reads covering the whole gene with 99-100% identity, confirming the exact source of this sample. However, in order to exclude contaminants we further blasted using *Drosophila mauritiana* COI *(*AF200831.1) because the putative new genome for *Caenorhabditis remanei* has 98% identity to wNo of *Drosophila mauritiana* in our Fig. 2b. Indeed, we found reads covering a portion (not all) of the gene with 98-100% identity, indicating a contamination from a *Drosophila mauritiana* or from another closely related *Drosophila*.

4. Host "tags" used to search the NCBI SRA repository

insects, insect, bug, bugs, worm, roundworm, roundworms, silkworm, armyworm, termite, termites, ant, ants, mite, mites, ticks, tick, springtail, springtails, bees, bee, wasp, wasps, flea, fleas, moth, moths, Beetle, Beetles, spider, spiders, Wolbachia, Arthropods, Arthropod, Nematodes, Nematode, Anopheles, Aedes, Folsomia, Culex, Ctenocephalides, Onchocerca, Brugia, Osmia, Drosophila, Armadillidium, Diaphorina, Dirofilaria, crickets, Caenorhabditis, Aedes, Agelenopsis, Anastrepha, Anax, Anoplolepis, Argynnis, Autographa, Azteca, Bemisia, Brugia, Caleta, Camponotus, Cimex, Colias, Coptotermes, Crematogaster, Culex, Danaus, Diaphorina, Dirofilaria, Encarsia, Ephestia, Erebia, Euphydryas, Eurema, Euwallacea, Formica, Glossina, Gryllus, Hypolimnas, Incisitermes, Ischnura, Junonia, Kerria, Laodelphax, Leptopilina, Limenitis, Lycaeides, Melanitis, Melitaea, Minois, Monomorium, Myrmecorhynchus, Myrmica, Nasonia, Neptis, Nilaparvata, Notoncus, Odontomachus, Odontotermes, Onchocerca,,thetrum, Ostrinia, Pantala, Papilio, Parantica, Parasite mite, Parnassius, Pheidole, Plebejus, Polistes, Polyergus, Polygonia, Polyommatus, Polyrhachis, Pseudomyrmex, Rhagoletis, Rhytidoponera, Sitona, Sogatella, Solenopsis, Spodoptera, Stenamma, Steriphus, Teleogryllus, Tetranychus, Tetrastichus, Tribolium, Trichogramma, Wasmannia, Xyleborus, Ypthima, Zootermes, cricket, mite, mites, Hexapoda, Collembola, Hexapoda, Isoptera, Zootermes, angusticollis, planthopper, leafhopper, butterfly, butterflies, crustacean, weevil, weevils, Ixodes, scorpion, scorpions, Acari, Acarina, cockroach, Cryptocercus, Cryptotermes, Termitidae, Alyscotermes, Macrotermitinae, Macrotermes, Microtermes, Odontotermes, Nasutitermitinae, Nasutitermes, Trinervitermes, Cornitermes, Syntermes, Termitinae, Amitermes, Amitermes, Globitermes, Microcerotermes, Cubitermes, Cubitermes, Ophiotermes, Neocapritermes, Promirotermes, Termes, "water bears", tardigrade, harvestman, symphylans, millipede, amphipod, isopod, oniscus, woodlice, carpenter, slater, simon, coneheads, Proturans, Diplurans, bristletails, silverfish, Mayflies, Dragonflies, damselflies, Grasshoppers, crickets, Earwigs, Stoneflies, Mantids, Cockroaches, cicadas, leafhoppers, aphids, psyllids, thrips, Booklice, Lice, Lacewings, Caddisflies, Caddisfly, Scorpionflies, Tardigrada, Onychophora, Chilopoda, Nematomorpha, Diplopoda, Symphyla, Arachnida, Amblypygi, Araneae, Opiliones, Pseudoscorpiones, Ricinulei, Scorpiones, Solifugae, Thelyphonida, Homoptera, Branchiopoda, Phyllopoda, Sarsostraca, Remipedia, Maxillopoda, Thecostraca, Branchiura, Copepoda, Ostracoda, Myodocopa, Malacostraca, Hoplocarida, Eumalacostraca, Protura, Diplura, Microcoryphia, Thysanura, Ephemeroptera, Odonata,,thoptera, Phasmatodea, Grylloblattodea, Mantophasmatodea, Dermaptera, Plecoptera, Embiidina, Zoraptera, Mantodea, Blattodea, Hemiptera, Heteroptera, Sternorrhyncha , Thysanoptera, Psocoptera, Phthiraptera, Amblycera, Anoplura, Coleoptera, Neuroptera, Hymenoptera, Trichoptera, Lepidoptera, Siphonaptera, Mecoptera, Strepsiptera, Diptera, crayfish, crab, crabs, “water bears", tardigrade, harvestman, symphylans, millipede, amphipod, isopod, oniscus, woodlice, carpenter, slater, simon, coneheads, Proturans, Diplurans, bristletails, silverfish, Mayflies, Dragonflies, damselflies, Grasshoppers,

crickets, Earwigs, Stoneflies, Mantids, Cockroaches, cicadas, leafhoppers, aphids, psyllids, thrips, Booklice, Lice, Lacewings, Caddisflies, Caddisfly, Scorpionflies, Tardigrada, Onychophora, Chilopoda, Nematomorpha, Diplopoda, Symphyla, Arachnida,OR Amblypygi, Araneae, Opiliones, Pseudoscorpiones, Ricinulei, Scorpiones, Solifugae, Thelyphonida, Homoptera, Branchiopoda, Phyllopoda, Sarsostraca, Remipedia, Maxillopoda, Thecostraca, Branchiura, Copepoda, Ostracoda, Myodocopa, Malacostraca, Hoplocarida, Eumalacostraca, Protura, Diplura, Microcoryphia, Thysanura, Ephemeroptera, Odonata,,thoptera, Phasmatodea, Grylloblattodea, Mantophasmatodea, Dermaptera, Plecoptera, Embiidina, Zoraptera, Mantodea, Blattodea, Hemiptera, Heteroptera, Sternorrhyncha , Thysanoptera, Psocoptera, Phthiraptera, Amblycera, Anoplura, Coleoptera, Neuroptera, Hymenoptera, Trichoptera, Lepidoptera, Siphonaptera, Mecoptera, Strepsiptera, Diptera

5. Keywords searched but not present in NCBI

Aciagrion, Acisoma, Acraea, Acromis, Aenictus, Aganaspis, Amblyptilia, Anthene, Apanteles, Aphantopus, Apoica, Araschnia, Ariadne, Aricia, Asobara, Azanus, Barronopsis, Brachythemis, Brachythems, Brangas, Cabera, Carcharodus, Carpomya, Carterocephalus, Castalius, Catopsilia, Celastrina, Cepora, Ceriagrion, Ceutorhynchus, Chelonus, Chelymorpha, Clossiana, Coenonympha, Colotis, Corcyra, Cordylochernes, Crocothemis, Cupido, Delias, Diaphorencyrtus, Dictyophara, Diplacodes, Diplazon, Doryctobracon, Dorymyrmex, Epophthalmia, Eretmocerus, Eriborus, Eumedonia, Evagetes, Everes, Fabriciana, Gambrus, Geometra, Glaucopsyche, Heodes, Heteropterus, Hipparchia, Hirtodrosophila, Horaga, Hylyphantes, Hyponephele, Hyposoter, Ictinogomphus, Iraota, Ixias, Jalmenus, Jamides, Lasiommata, Leptidea, Leptogenys, Leptomyrmex, Leptosia, Leucophenga, Libythea, Lissorhoptrus, Lophomyrmex, Lopinga, Lycaena, Macrosteles, Maculinea, Mansonia, Melanargia, Metapone, Microgaster, Muscidifurax Nacaduba, Neurothemis, Nymphalis, Ochetellus, Ochlodes, Ocymyrmex, Odontosema, Opistograptis, Opistophthalmus, Opius,,eina,,ius,,nipholidotos,, Pareronia, Petrobia, Pleuroptia, Polybia, Pontia, Protocalliphora Pseudozizeeria, Pyrgus, Satyrium, Slavum, Spalgis, Sueus, Suillia, Surendra, Sycoscapter, Syrphophilus, Tarucus, Technomyrmex, Telicada, Teractrocera, Thecla, Thersamonia, Tongeia, Trithemis, Tymmophorus, Udaspes, Walkerella, Xylosandrus, Zizeeria, Demodex, Acherongia, Acherontides, Acherontiella, Acheroxenylla, Austrogastrura, Barbagastrura, Biscoia, Bonetogastrura, Celegastrura, Ceratophysella, Choreutinula, Cosberella, Denigastrura, Ecuadogastrura, Gnathogastrura, Gomphiocephalus, Hypogastrura, Jacutogastrura, Mesachorutes, Mesogastrura, Microgastrura, Neobeckerella, Octoacanthella, Ongulogastrura,,ogastrura, Parawillemia, Paraxenylla, Pseudacherontides, Schaefferia, Stenogastrura, Tafallia, Taurogastrura, Thibaudylla, Triacanthella,OR Typhlogastrura, Willemgastrura, Willemia, Xenylla, Xenyllogastrura, Hypogastruridae

Supplementary Figures

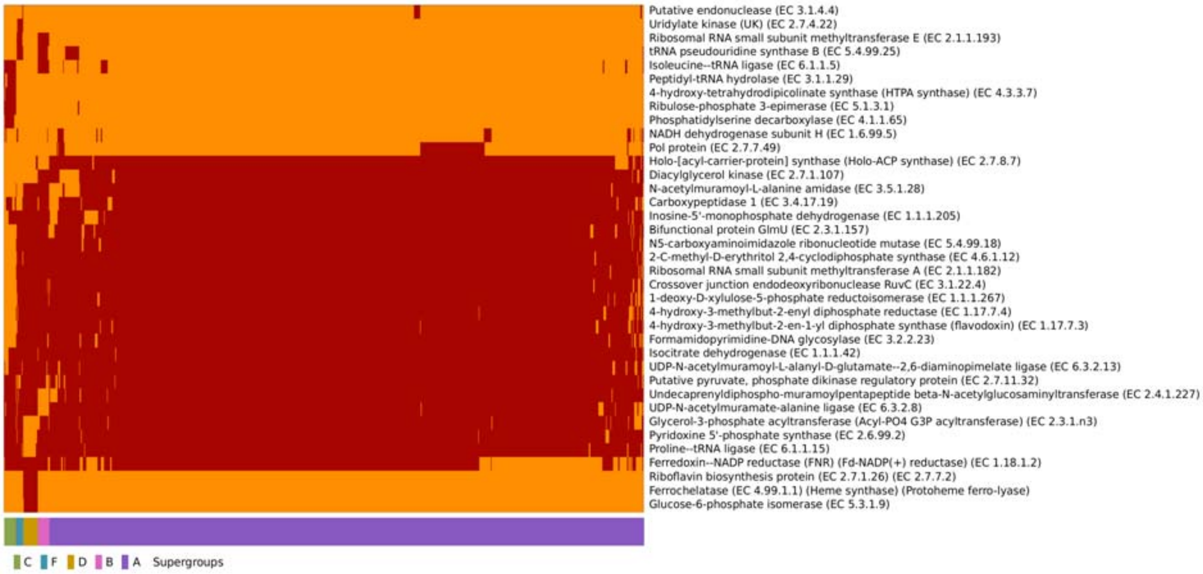

Supplementary Figure 1. Heatmap of the comparative genomic analysis of 989 novel high quality *Wolbachia* assemblies associated to the 14 host groups in Fig. 4b. Shown are enzyme categories (EC) that are significantly different between host groups (Fisher test).

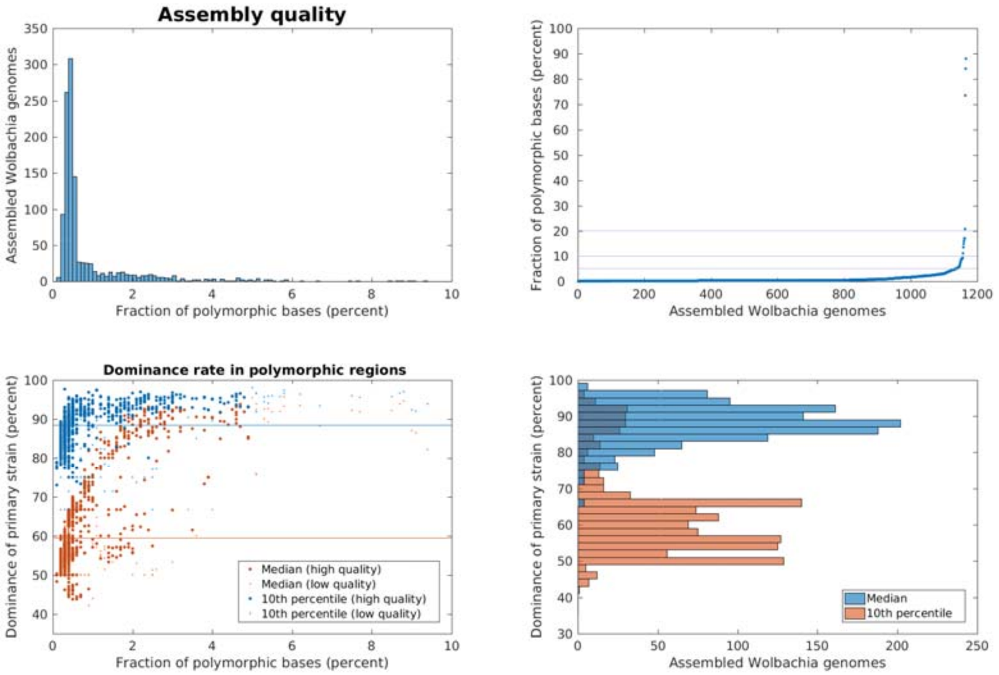

Supplementary Figure 2. Assembly quality control based on polymorphic site identification. (**a,b**) Most assembled *Wolbachia* genomes show polymorphic pattern in less than one percent of the total genome size. (**c,d**) In these polymorphic regions, the primary bases show a median dominance of 88.3 percent. In the lower 10th percentile, the median dominance of the primary sequence is 59.45 percent (horizontal lines). Based on four criteria including polymorphism, assembled genomes are considered as of high quality (big dots), low quality (small dots).

Supplementary Figure 3: Co-phylogenies of *Wolbachia* and mitochondria reconstructed from the same Sequence Read Archive file for 11 different host species (in 15 different panels). Trees are the posterior consensus and values at nodes are posterior probabilities from BEAST analysis employing for all analyses GTR+G replacement model, strict uncalibrated clock, and constant coalescent tree priors. Plus in some cases Bootstrap supports from a GTR+G RAxML analysis. For *D. melanogaster* and *D. simulans* we further show a cladogram of the BEAST tree to ease discriminating nodes and supports.

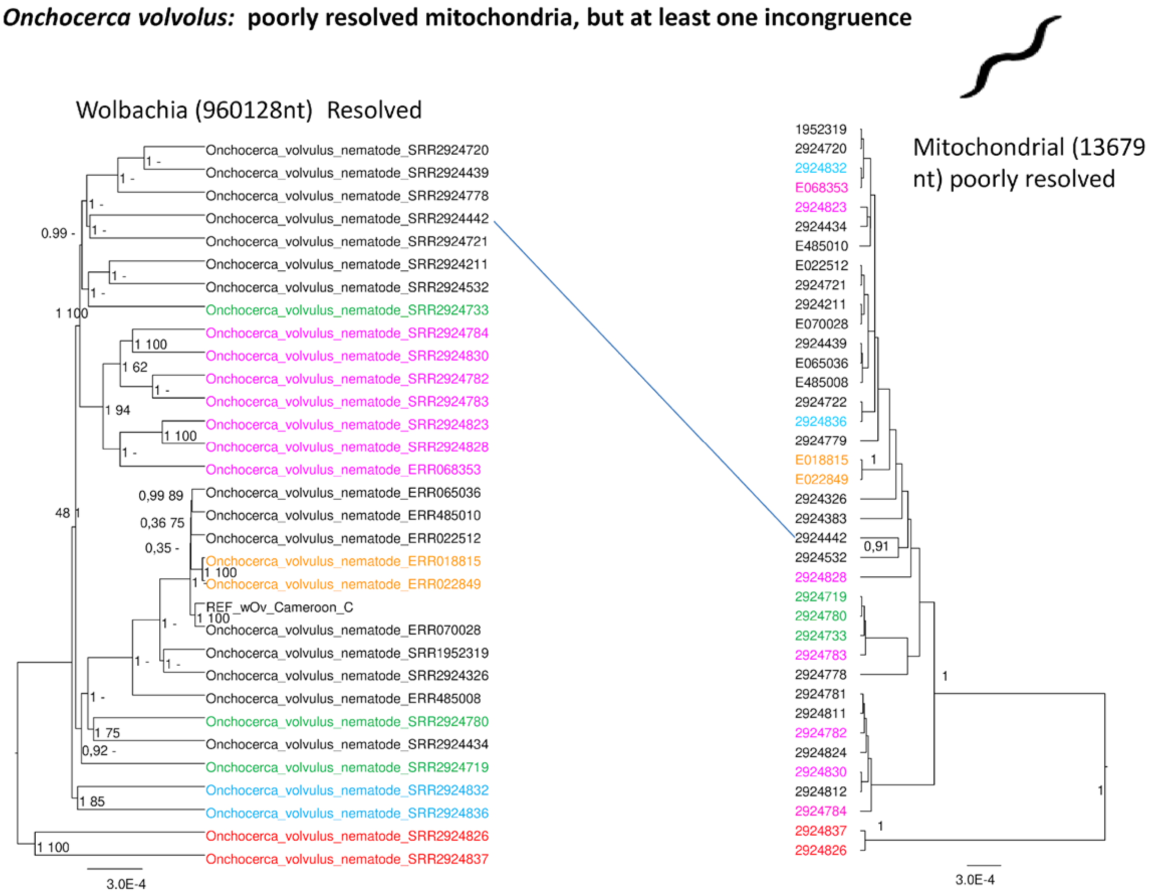

***Onchocerca ochengi*: well resolved topologies and at least 2 well supported incongruencies**

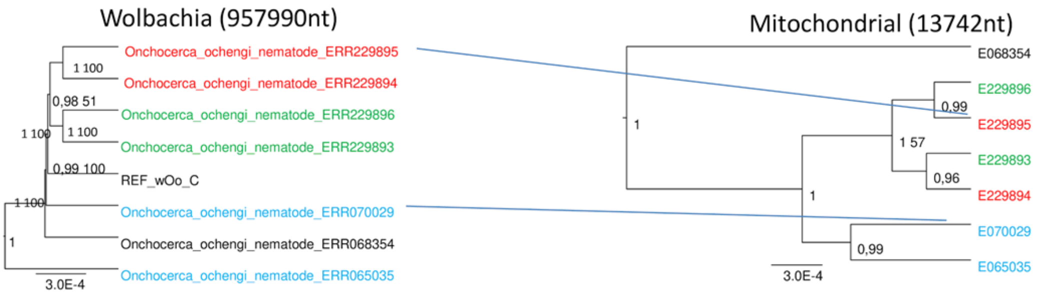

***Cimex lectularius*: partially resolved mitochondria, at least four incongruencies**

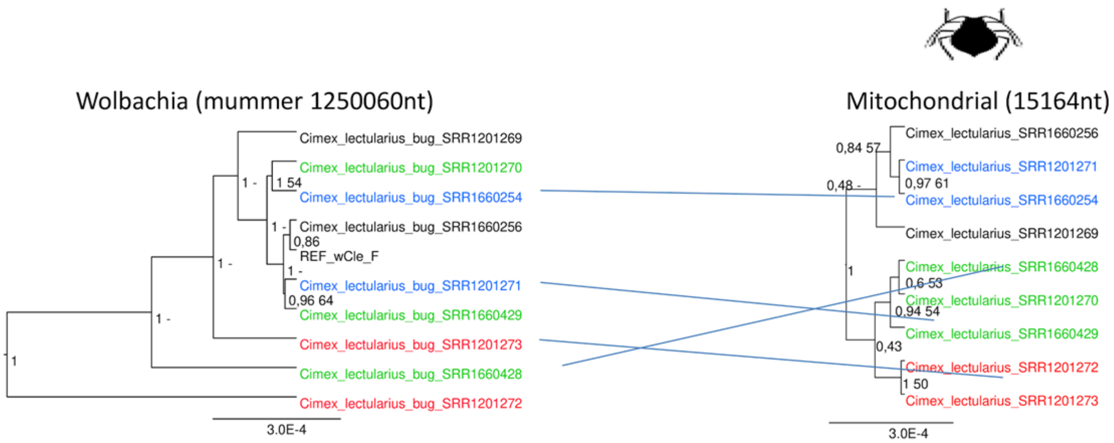

***Brugia malayi:* non significant incongruence due to unresolved mito phylogeny**

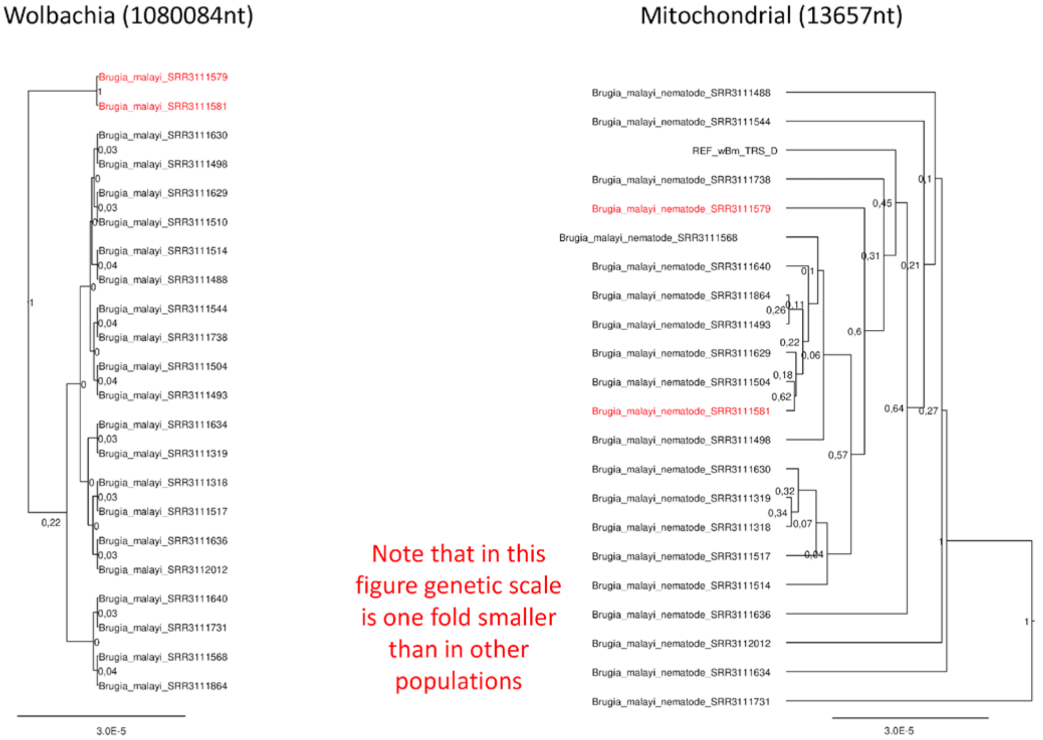

***Diabrotica virgifera:* partially resolved mitochondria, at least three incongruences**

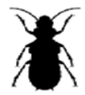

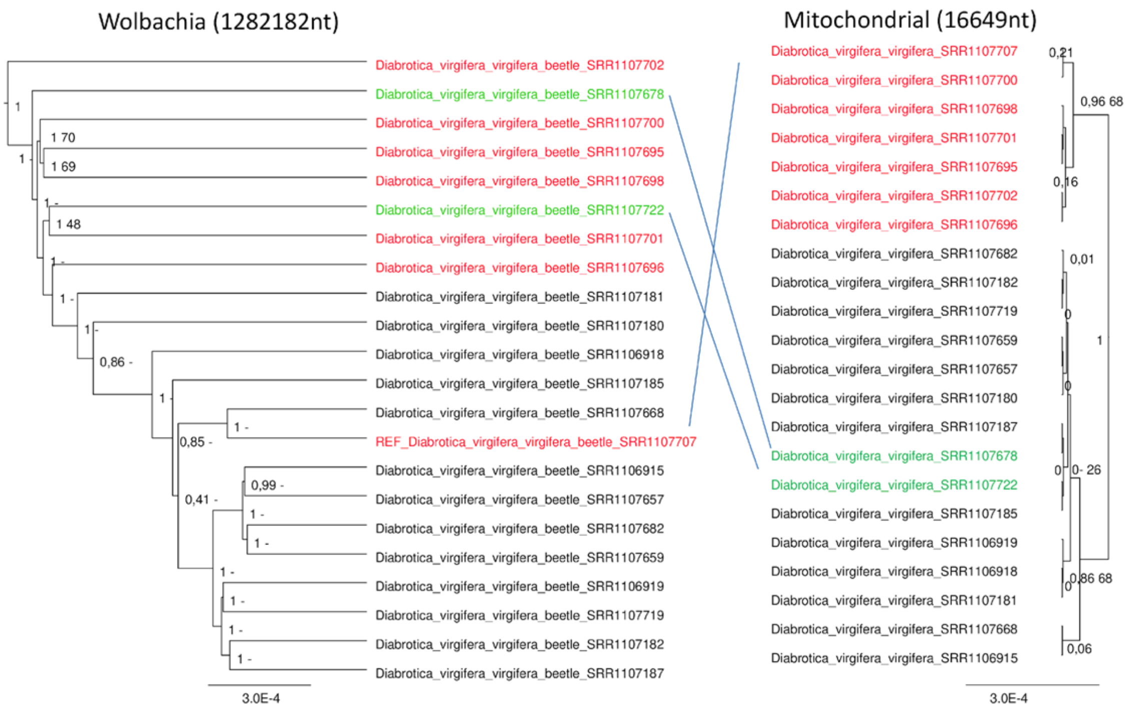

***Diaphorina citri:* *un*resolved (identical) mitochondria**

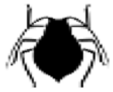

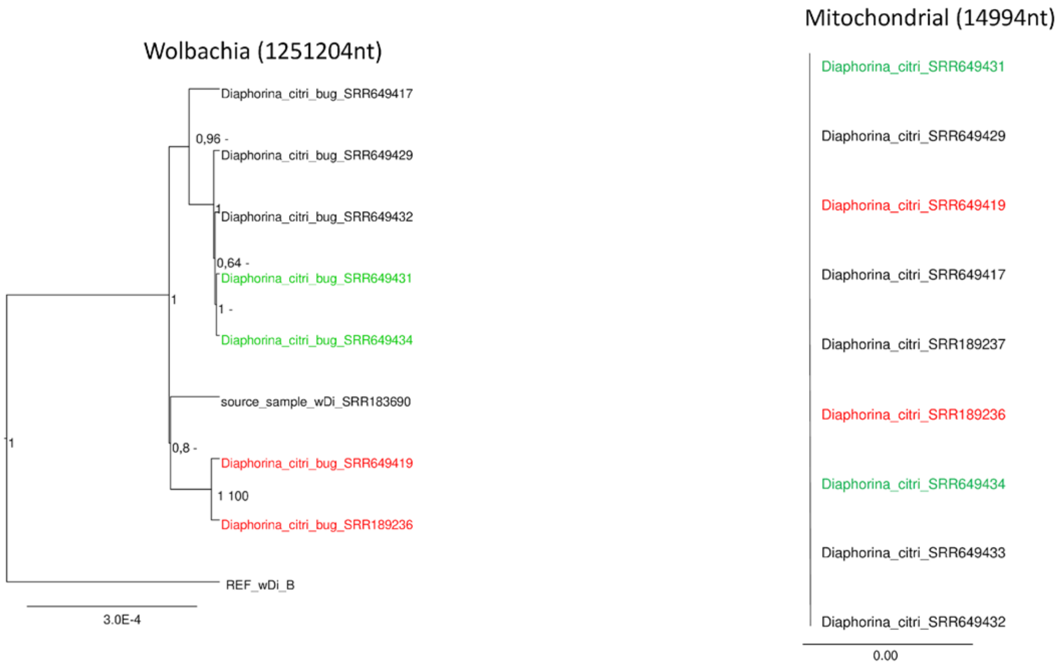

D.ananassae: **MANY well supported incogruences.**

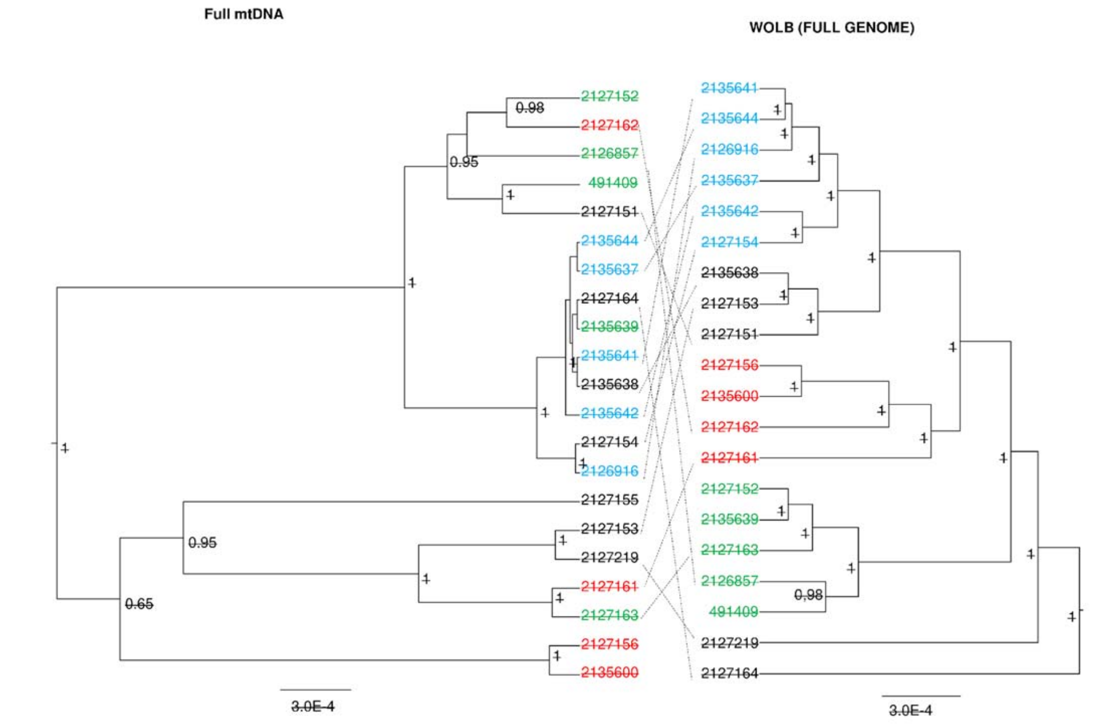

D. mauritiana: **one (poorly supported) incongruences**

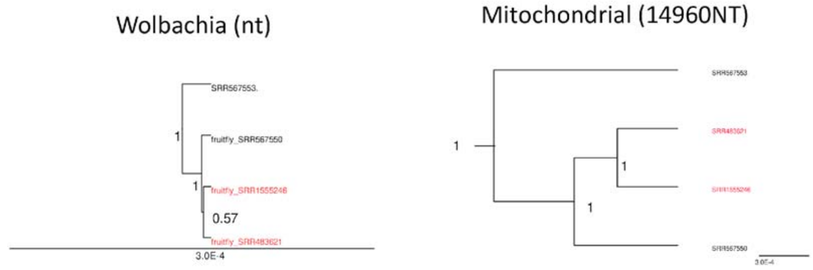

***Diachasma alloeum*: unresolved mitochondria, one incogruence due to well supported root**

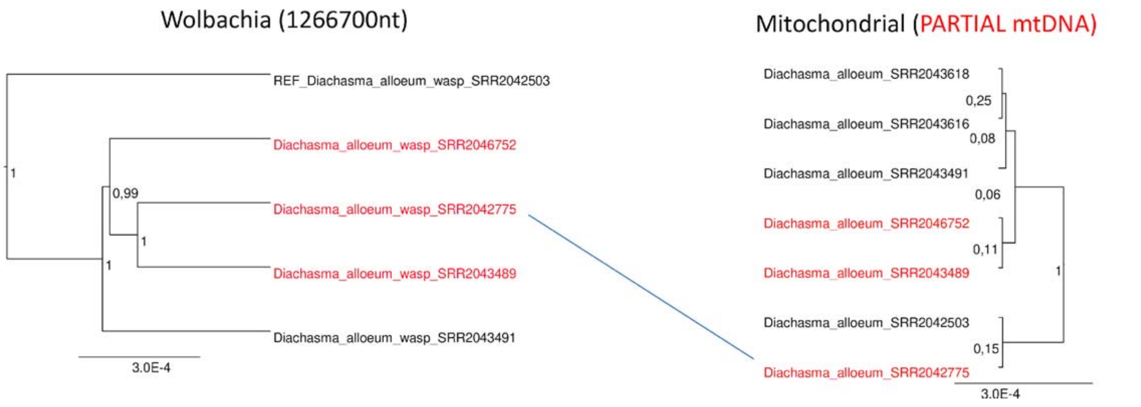

***D. melanogaster:* BEAST CLADOGRAM, partially resolved, but various incogruences**

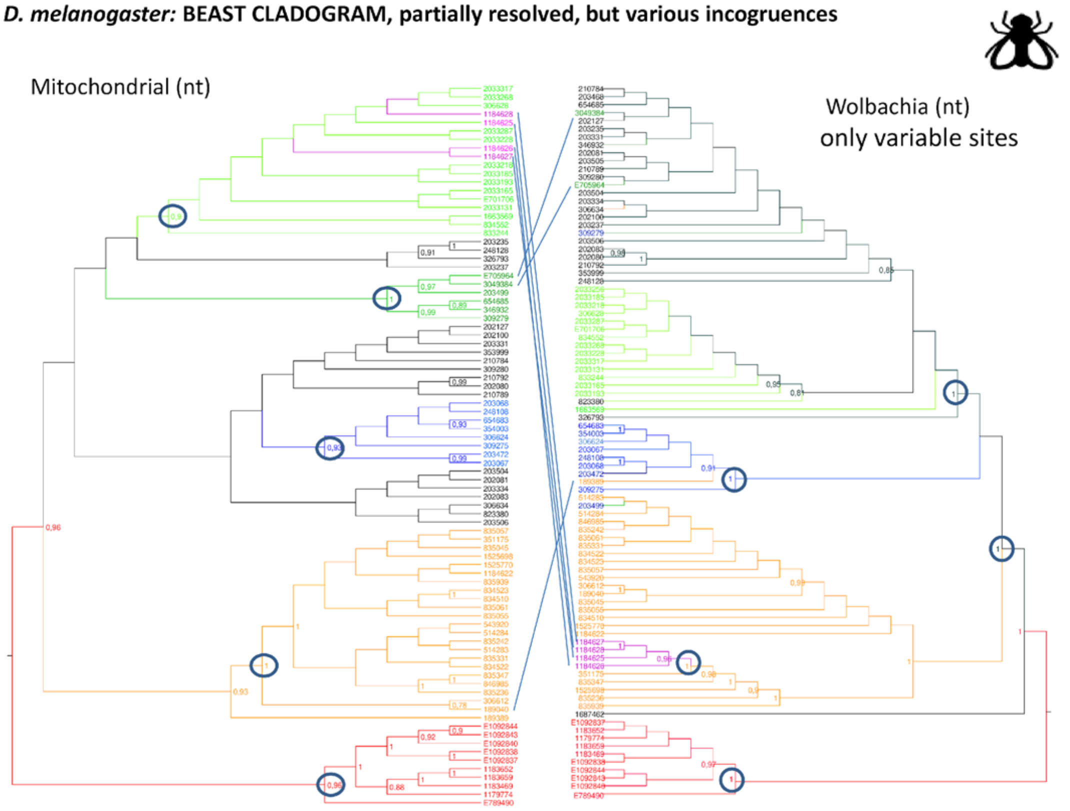

***D. simulans*: BEAST cladogram, moslty unresolved at least one incongruence**

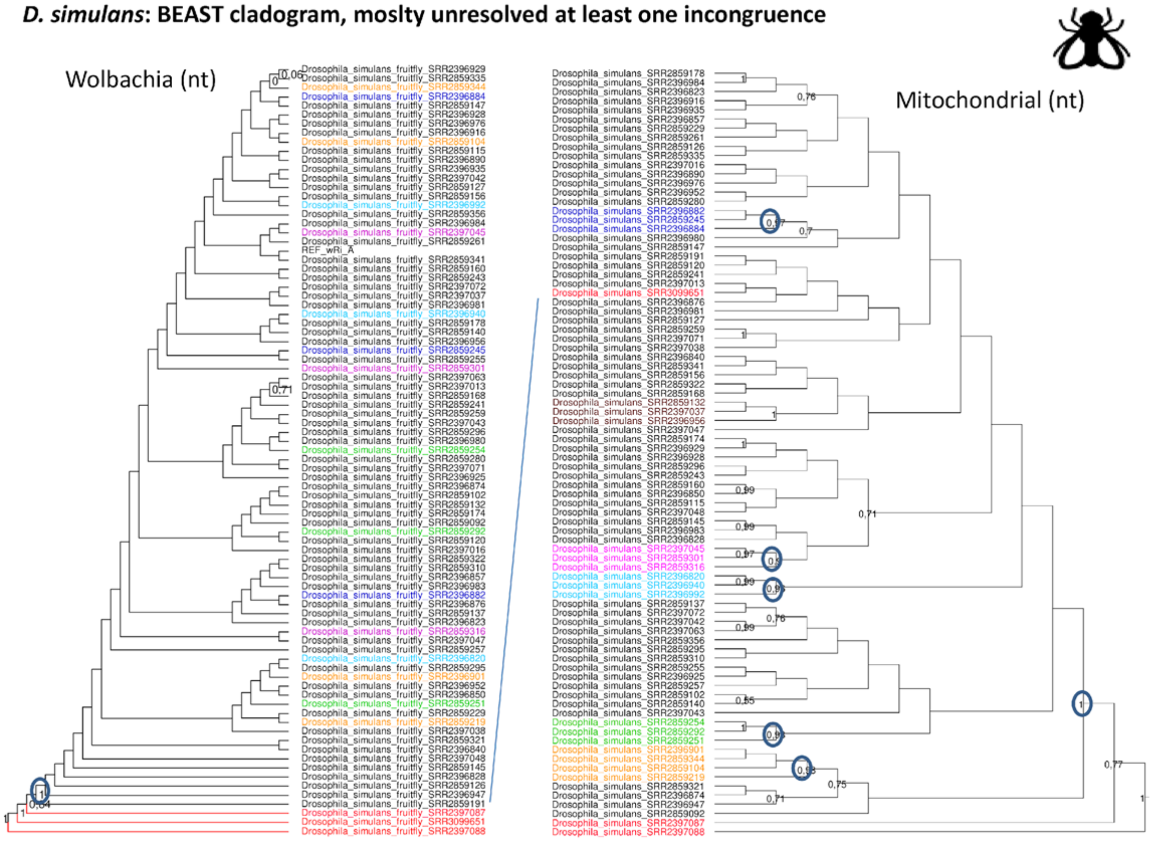

D. melanogaster: **original BEAST trees with actual branch lenghts.**

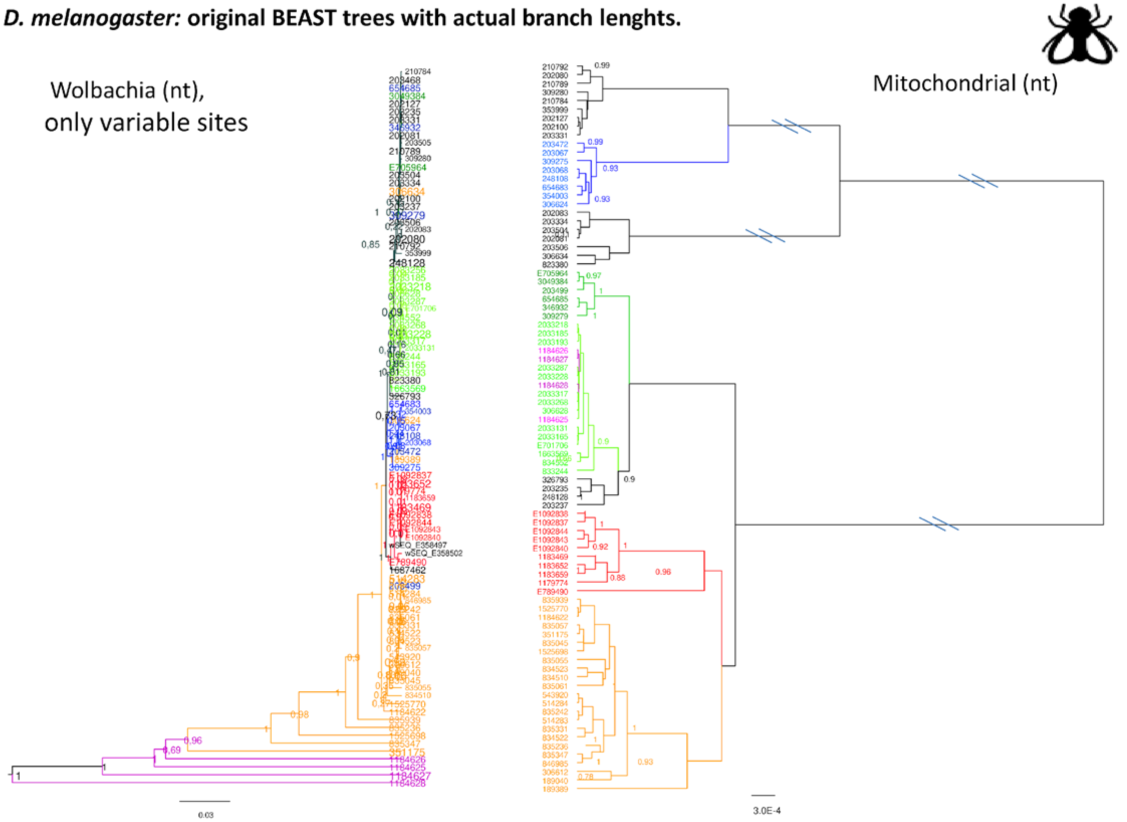

D. simulans **: original BEAST trees with actual branch lenghts**

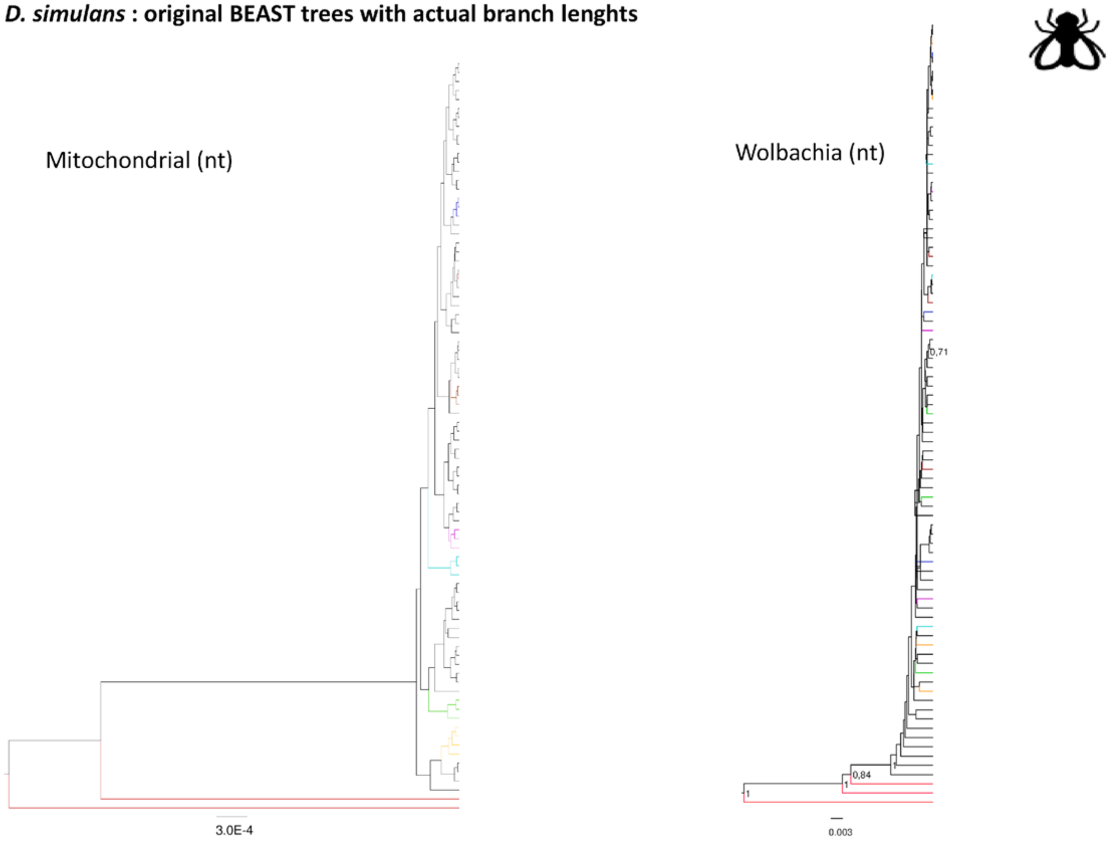

D. melanogaster , BEAST rescaling using Raxml tree lenght

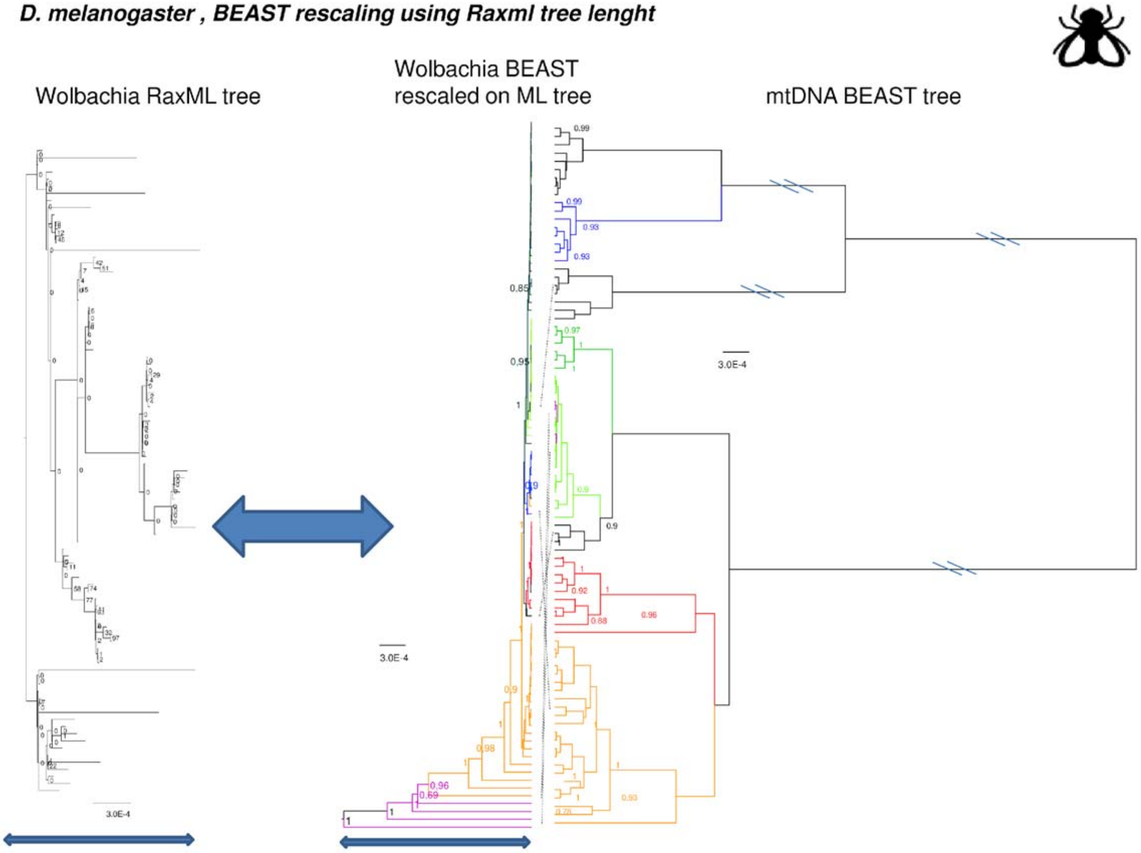

D. simulans, BEAST rescaling using Raxml tree lenght

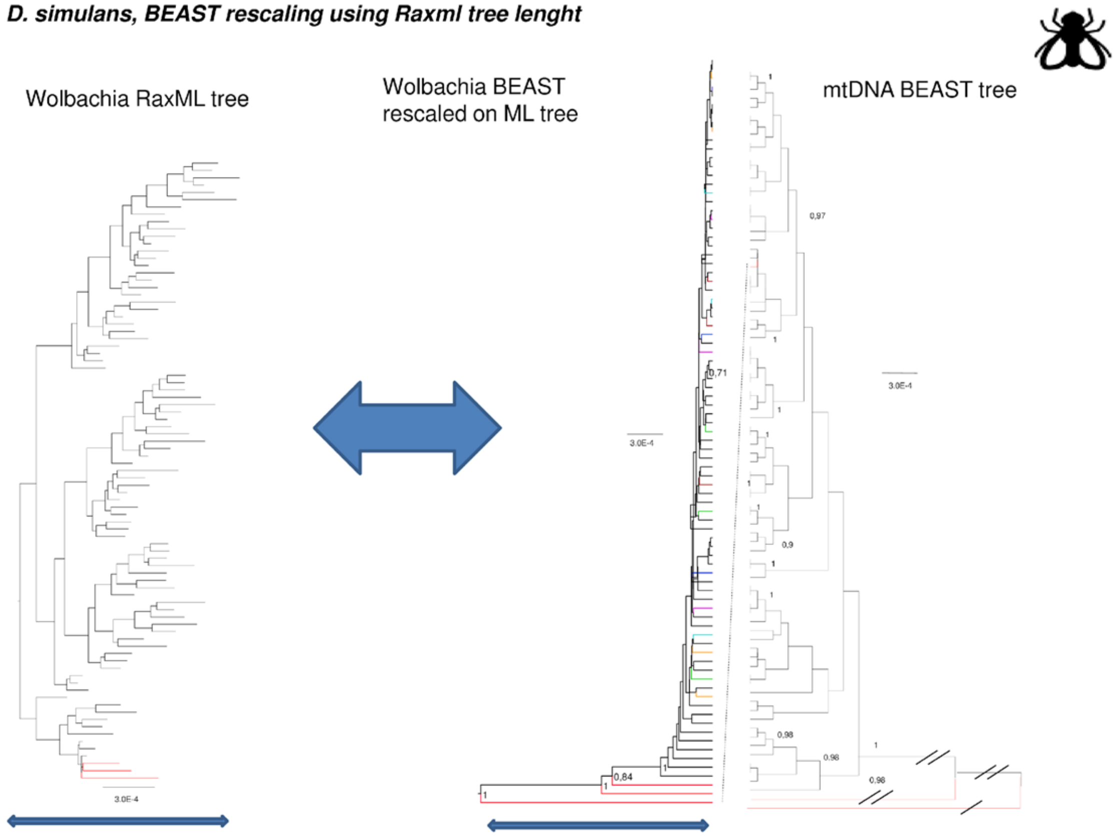

Supplement: Supplementary file 1 — Supplementary Information [file 41467_2020_19016_MOESM1_ESM.pdf]
