# Supplementary material for: Large scale genome reconstructions illuminate Wolbachia evolution
Source: Nat Commun. 2020 Oct 16;11:5235. doi: 10.1038/s41467-020-19016-0 (PMC7568565; doi:10.1038/s41467-020-19016-0)
Supplement: Supplementary file 3 — Reporting Summary [file 41467_2020_19016_MOESM3_ESM.pdf]

nature research

Corresponding author(s): Omar Rota Stabelli

Last updated by author(s): Apr 26, 2019

Reporting Summary

Nature Research wishes to improve the reproducibility of the work that we publish. This form provides structure for consistency and transparency in reporting. For further information on Nature Research policies, see Authors & Referees and the Editorial Policy Checklist.

Statistics

For all statistical analyses, confirm that the following items are present in the figure legend, table legend, main text, or Methods section.

n/a	Confirmed	
☐	☒	The exact sample size (*n*) for each experimental group/condition, given as a discrete number and unit of measurement
☐	☒	A statement on whether measurements were taken from distinct samples or whether the same sample was measured repeatedly
☐	☒	The statistical test(s) used AND whether they are one- or two-sided<br>*Only common tests should be described solely by name; describe more complex techniques in the Methods section.*
☐	☒	A description of all covariates tested
☐	☒	A description of any assumptions or corrections, such as tests of normality and adjustment for multiple comparisons
☐	☒	A full description of the statistical parameters including central tendency (e.g. means) or other basic estimates (e.g. regression coefficient) AND variation (e.g. standard deviation) or associated estimates of uncertainty (e.g. confidence intervals)
☐	☒	For null hypothesis testing, the test statistic (e.g. *F*, *t*, *r*) with confidence intervals, effect sizes, degrees of freedom and *P* value noted<br>*Give P values as exact values whenever suitable.*
☐	☒	For Bayesian analysis, information on the choice of priors and Markov chain Monte Carlo settings
☒	☐	For hierarchical and complex designs, identification of the appropriate level for tests and full reporting of outcomes
☒	☐	Estimates of effect sizes (e.g. Cohen's *d*, Pearson's *r*), indicating how they were calculated

Our web collection on statistics for biologists contains articles on many of the points above.

Software and code

Policy information about availability of computer code

Data collection	Download from NCBI Sequence Read Archive (SRA) was performed using the sratoolkit (prefetch and fastq-dump) version 2.8.2
Data analysis	Bowtie2 version 2.1.0; Samtools version 1.3.1; PanPhlAn version 1.2; MegaHit version 1.0.5; Prokka version 1.11; Roary version 3.5.9; BLAST version 2.8.1; Mafft version 7.215; RAxML version 8.2.9, Beast version 1.8.

For manuscripts utilizing custom algorithms or software that are central to the research but not yet described in published literature, software must be made available to editors/reviewers. We strongly encourage code deposition in a community repository (e.g. GitHub). See the Nature Research guidelines for submitting code & software for further information.

Data

Policy information about availability of data

All manuscripts must include a data availability statement. This statement should provide the following information, where applicable:

- Accession codes, unique identifiers, or web links for publicly available datasets
- A list of figures that have associated raw data
- A description of any restrictions on data availability

All assembled Wolbachia genomes can be accessed at https://sites.google.com/fmach.it/wolbachia

Field-specific reporting

Please select the one below that is the best fit for your research. If you are not sure, read the appropriate sections before making your selection.

☐ Life sciences ☐ Behavioural & social sciences ☒ Ecological, evolutionary & environmental sciences

Ecological, evolutionary & environmental sciences study design

All studies must disclose on these points even when the disclosure is negative.

Study description	Large screen of over 30,000 publicly available shotgun sequencing samples in order to expand the number of Wolbachia genomes across host phylogenies.
Research sample	Public available NCBI-SRA sequencing samples of potential Wolbachia arthropod and nematode hosts
Sampling strategy	NCBI database search based on 181 arthropod and nematode host related keywords
Data collection	Download of public available NCBI-SRA sequencing samples
Timing and spatial scale	Sample download: January to July 2017
Data exclusions	No data were excluded
Reproducibility	Results are reproducible using the computational tools as described in the Method section
Randomization	Samples are grouped by host species based on NCBI metadata
Blinding	Not relevant

Did the study involve field work? ☐ Yes ☒ No

Reporting for specific materials, systems and methods

We require information from authors about some types of materials, experimental systems and methods used in many studies. Here, indicate whether each material, system or method listed is relevant to your study. If you are not sure if a list item applies to your research, read the appropriate section before selecting a response.

Materials & experimental systems

n/a	Involved in the study
☒	☐ Antibodies
☒	☐ Eukaryotic cell lines
☒	☐ Palaeontology
☒	☐ Animals and other organisms
☒	☐ Human research participants
☒	☐ Clinical data

Methods

n/a	Involved in the study
☒	☐ ChIP-seq
☒	☐ Flow cytometry
☒	☐ MRI-based neuroimaging
