# Supplementary material for: Large scale genome reconstructions illuminate Wolbachia evolution
Source: Nat Commun. 2020 Oct 16;11:5235. doi: 10.1038/s41467-020-19016-0 (PMC7568565; doi:10.1038/s41467-020-19016-0)
Supplement: Supplementary file 4 — Description of Additional Supplementary Files [file 41467_2020_19016_MOESM4_ESM.docx]

**Description of Additional Supplementary Files**

File name: Supplementary Data 1

Description: “Scholz_CoreSeqTree of Fig2a_RAxML_bipartitions_313coreGenes.nwk” is the Maximum likelihood tree (Fig. 2a) of 1161 newly assembled Wolbachia genomes (see Supplementary Data 5). The tree includes bootstrap support values and is provided in Newick format.

File name: Supplementary Data 2

Description: the co-phylogenetic trees of both Wolbachia and host mitochondria (mtDNA) used to generate Fig. 4 and Supplementary Fig. 3. For each tree we provide the tree in Figtree format and the xml file used ot generate it in BEAST.

File name: Supplementary Data 3

Description: Wolbachia reference genomes. List of 43 public available Wolbachia reference genomes, including NCBI accession number, basic genomic parameters, and literature reference.

File name: Supplementary Data 4

Description: Wolbachia prevalence. Number of downloads per host. Prevalence of Wolbachia positive samples at PanPhlAn and assembly level. Wolbachia-free host species with less than 50 downloads are excluded.

File name: Supplementary Data 5

Description: Assembled Wolbachia genomes, including quality check and host identity using 18S. Table describes all 1161 assembled Wolbachia genomes of the core gene tree (Fig. 2a), including 1005 high quality and 156 tree quality assemblies. Several assembly quality measures are provided, including polymorphic rates, dominance of the primary strain, and genome length, which in combination define whether an assembly is considered as being of high, low quality (see Methods).

File name: Supplementary Data 6

Description: Host specific functional gene gain and loss. Table provides significant gene gain and loss (Fisher test) of 14 host specific Wolbachia populations and 11 candidate genes that are significantly enriched in Cytoplasmic Incompatibility (CI) associated lineages. Functional differences are reported at gene-family level, KEGG and at enzyme categories (EC).

File name: Supplementary Data 7

Description: The average divergences over all sequence pairs used for Fig. 4b-c
