# Supplementary material for: Large scale genome reconstructions illuminate Wolbachia evolution
Source: Nat Commun. 2020 Oct 16;11:5235. doi: 10.1038/s41467-020-19016-0 (PMC7568565; doi:10.1038/s41467-020-19016-0)
Supplement: Supplementary file 2 — Peer Review File [file 41467_2020_19016_MOESM2_ESM.pdf]

Reviewers' comments:

Reviewer #1 (Remarks to the Author):

Wolbachia is the most common and arguably one of the most important endosymbionts – and it certainly has received a fair share of attention. Quite surprisingly, for a bacterium with a relatively small genome size, genomic data is still sparse and heavily biased towards a few taxonomic groups. Several times, Wolbachia was discovered as sequencing by-product in studies aiming to sequence its host, but there never has been an attempt at systematically screening *all* short read data from potential hosts (nematodes + arthropods). Scholz et al must be commended for undertaking this stupendous task, and the resulting dataset of >1000 genomes will undoubtedly be very useful to the Wolbachia community. There are two examples given of what kind of questions one can tackle with such a dataset. Probably the most exciting finding (and this may be personal bias) is that Wolbachia evolutionary rates vary a lot between hosts, and that this also varies with host life history traits. This seems very plausible and logical, yet most (even very recent) publications assume that the rate determined by Richardson et al in Drosophila is fixed throughout all Wolbachia/host combinations. Its really nice to have a strong dataset now that demonstrates the variability in Wolbachia evolutionary rates – although I do think that some additional data is necessary to support this claim (see below).

I would very much like to see this paper published, but I want to ask for a few clarifications before I can recommend acceptance of the manuscript – in the hope that this will improve the paper.

Major points:

1) Please provide clarification on the number of truly novel genomes that were reconstructed. It seems that many genomes are almost identical to the already published wMel and wRi variants, and others have likely already been described, e.g., in Richardson et al. (https://doi.org/10.1371/journal.pgen.1003129), Turelli et al. (https://doi.org/10.1016/j.cub.2018.02.015), or Choi et al. (https://doi.org/10.1038/nmicrobiol.2016.207). Also, how many of the novel genomes were identical? Related to this: for novel data from already sequenced genomes (wMel, wRi), wouldn't it have made more sense to simply call variants instead of assembling from scratch? The chance for errors is probably higher when trying to assemble denovo.

2) One problem with reconstructing genomes from such published data is the lack of quality checks – your inference is only as accurate as the metadata associated with the SRA data. I wonder if you could attempt to verify the host species for each of the novel Wolbachia genomes; for example by filtering out the COI gene and blasting that.

3) In your functional analysis, non-CI strains are compared with CI strains, and significantly enriched genes are identified as candidates. This is problematic as there is no phenotypic data for most of the analysed strains. If you make the assumption that all genomes that are very similar to the ones causing CI also cause CI, your argumentation becomes circular: first you group the genomes by similarity and then you identify features that make them similar. Also, I think there may be a systematic biased introduced in testing for enrichment by including this many wMel and wRi variants which are essentially identical in gene content. Further, the you should compare the genes that you have identified with the candidates identified through comparative genomics in LePage et al (https://doi.org/10.1038/nature21391), there may be some overlap. Finally, if you find phage genes significantly enriched, this is not that surprising, as the CI loci are located in a prophage region.

4) I think to demonstrate that Wolbachia evolution is indeed as heterogenenous as claimed, one might also have to look at nuclear data. If you only compare rates of mitochondria and Wolbachia, it could also be that it's the mitochondria that actually evolve at very different speeds between taxa (which is not totally unreasonable to assume), whereas the Wolbachia might evolve ~equally fast. It's a very

good dataset to test this– you should try and extract a couple of housekeeping genes that are present in all of the hosts and determine their evolutionary rates.

5) Please upload the novel genomes to a permanently accessible database, such as Datadryad – its not that expensive (only ~2% of the Nature Communications APC) and will ensure long term access for the community.

Minor points & suggestions, hopefully constructive:

Title
Suggest to delete 'Large scale'

Line 48
There may be only 43 reference genomes, but if you define genomes as you define them in the title of your manuscript, the number is substantially larger (see major comment 1).

Line 57
Suggest to change 'because of no phylogenetic signal' to 'due to lack of phylogenetic signal'

Figure 1
This should be 2 separate Figures, 2b is barely readable.

Line 111
I couldn't find information on how the host genome size was determined. Are all estimates from the literature?

Line 168–169
These genomes are published: https://doi.org/10.1101/461574 and http://dx.doi.org/10.1101/551036

Line 177
Various bees -> 2 bee species (according to your Table S3)

Line 178
Thetranycus urticae -> Tetranychus urticae

Line 195–198
These are examples of why it would be good to verify the host species with COI or similar. For example, Holcocephala fusca is a robber fly and thus predatory. Depending on the DNA prep it is very possible that this Wolbachia strain is from a prey individual, rather than from the robber fly. Also, without demonstration that there is no other potential host species in the sequencing data of Caenorhabditis remanei, I find it very difficult to believe that Wolbachia is actually present in this species. To my knowledge, this would be the first Wolbachia strain detected in the Rhabditidae – all other nematode-Wolbachia associations are limited to the filarial nematodes.

Line 205–206
I can't follow this line of argumentation: most Wolbachia from Hymenoptera in your supergroup A tree are not from parasitic lineages.

Line 222–224
This is not surprising, as most of the genomes from these groups would belong to highly adapted strains with degraded genomes.

Line 370
Please clarify “arthropod and nematode host related keywords”

Line 415ff
Please clarify: does this mean all contigs of one metagenomic assembly would have to match *the same* or *any* Wolbachia reference?

Line 428ff
Is number of contigs not considered important to call an assembly “high quality”?

Line 437ff
That’s a very cool approach to determine if multiple Wolbachia strains are present!

Line 474
“pident” -> “percent identity”

Line 476ff
In terms of alignment accuracy, it might make sense to align the loci separately and then concatenate. Maybe also exclude recombining loci?

Supplementary information

Line 24ff
Please avoid using “basal” to describe the position of taxa in trees.

Line ~128
“incongrences” -> “incongruencies”
“Cimex lecturalis” -> “Cimex lectularius”

There are 3 metagenomic samples in the dataset (2x "Apoidea", 1x "Insecta") – I wonder if it makes sense to include these here, as a host cannot be assigned.

Reviewer #2 (Remarks to the Author):

Schloz et al., review

Wolbachia is an important intracellular symbiont that has - and continues to impact insect populations and evolution. Since the sequencing of the first Wolbachia genome in 2004, there have been many efforts to increase sampling of Wolbachia across the genus (clades) and from different host species (orders of insects, other arthropods, nematodes, etc). Here, Schloz et al mine publicly available sequences from the SRA to identify possible Wolbachia sequences/reads based on mapping reads to available Wolbachia reference genomes (43 total currently). Their pipeline for identification of Wolbachia reads is straightforward and this approach (mapping to existing genomes) has been used by others. The authors claim to identify over 1,000 new Wolbachia genomes using this method - indeed, that would be a triumph and could provide a lot of interesting information about Wolbachia evolution and horizontal transfer between species. My main concern is the lack of caution used with regards to potential contamination from other bacteria (the microbiome) and from host lateral insertions (Wolbachia genomic fragments inserted into host nuclear genomes).

There are some major flaws, based on incorrect assumptions, in these data and their analyses.
First, Wolbachia are well known to integrate into their host genomes - so called “lateral gene transfer” or LGT. Many of the reads that the authors identify across insect species could come from these LGTs

and there is no validation of their data to account for robustness against LGTs. The authors set a threshold of 50% core genes for inclusion of these genomes but frankly, this is much too lenient. We know at least two cases where entire Wolbachia genomes are integrated into the host nuclear genome.

Also, they do not account for potential bacterial reads that map to the conglomerate Wolbachia assemblies just due to homology - this is indeed a major issue that we have seen many times in resequencing Wolbachia genomes.
Similarly, the number of polymorphic reads they allow in their assembly is very high for Wolbachia infection. I contest the use of "high quality" throughout — a "high quality" genome would have a less fragmented assembly. The fact that the authors could identify multiple paralogs of Wolbachia "core genes" by Blast within their "high quality" genomes should have been a red flag.

Also, there seems to be no curation of the SRA content before they run their assemblies - these datasets could come from pools of animals or environments, making downstream interpretations really difficult. How can you say that there is a 5% prevalence if the samples are pools of 100 flies, for example?

Finally, with regards to the bioinformatics, there is absolutely no benchmarking or validation on datasets KNOWN to be infected or uninfected. That would've provided better confidence. For example, the D. ananassae assembly has an integrated Wolbachia genome - how does their pipeline perform in the face of that?

More minor comments:

Line 124: "reduced genomes" or "shorter genomes relative to the other strains"

Reviewer #3 (Remarks to the Author):

Overall, I think the manuscript is well reasoned and written. The effort undertaken by the authors is large and the results seem equally impactful to the community at-large. I appreciate the succinct language and high quality of the writing – the manuscript was enjoyable to read. I have a few small comments/questions for the authors and then 2 larger concerns (one which should be easy enough for the authors to adjust).

Small concerns/typos/questions:
Ln 196-197. What method was used to determine the % nucleotide identity here? I could not find it in the methodology.
Ln 380. I was unclear as to what "pre-selected" referenced? I believe it's the 1,801 samples that had quantifiable Wolbachia signature?
Ln 458. How were representative selected?
Ln 471. pident is an abbreviation and should be explained for readers
Ln 504. In this instance does "sequences" mean "genomes"? If so, please change. If not, please clarify.
MUMmer assemblies. I understand the need to implement an alternative method when the datasets get large, so the switch to a MASH based approach for strains infecting D. melanogaster and wRi strains is logical. I just wonder the impact this has on the output presented in Figure 4. Is there any way of determining these results using the same methodology for all? Could the MASH approach be used for the smaller datasets and then any findings could more definitively linked to some underlying biological principle as opposed to possible influence of methodology?
Roary for pangenomes. I'm not personally not up on the current accepted understanding of Wolbachia

phylogeny, but in reviewing Roary it came to my attention that there are several warnings in the FAQ against using this tool for organisms outside of the same species. Now the Fig. 2 tree clearly illustrates that Wolbachia genomes from D. simulans could be considered strains of the same species, but my interpretation of the tree would be that the supergroups likely represent different "species" of Wolbachia. Possibly? The Roary settings used by the authors is 80% nucleotide identity. The Roary FAQ explicitly states that 70% would be too loose to be meaningful and recommends the lowest value of 90% identity for "diverse species". I wonder on what the results were for the authors when using the default and 90% cutoffs? And if the authors considered adjusting the mcl inflation value to influence clustering results? Determining where the species cutoff is for these groups may be aided (and would place these genomes in the context of other elements of the bacterial tree of life) by using the GTDB-Tk tool (https://github.com/Ecogenomics/GTDBTk).

Concerns:
(1) Storage of the genome results in a detected Google site is one way of making the data public, but I suggest that authors instead store the data in official repository that includes version tracking, DOIs, and robust legacy protocols, such as figshare or Dryad. While custom websites achieve this goal at bare minimum level, ensuring that the data is preserved in perpetuality should a website break or webmaster level, etc. should be main priority of making the data available, public, and reusable. I would recommend the authors review the FAIR practices for data availability (https://www.nature.com/articles/sdata201618).

(2) This concern has to do with how the new Wolbachia genomes fit in to the current language and accepted practices for metagenome-assembled genomes (MAGs). It is clear that the "genomes" for this manuscript are in fact, as the polymorphic data presented in Supplemental Table 3 confirms, MAGs and consist of a population-level genome-equivalents. The authors should make it clear to the readers that these genomes are not pure isolate genomes and therefore should not be treated as such.
To further address this, the authors need to put their genomes and definition of "high-quality" in the same context as other MAG datasets and the practices detailed by Bowers et al. (https://www.nature.com/articles/nbt.3893). Wolbachia may be a great edge case as to why these standards may not be appropriate, but these guidelines should be adhered to, if possible, and acknowledge and explained why if they cannot be. If not possible, I would recommend that the authors develop similar standards to Bowers et al. that are logical for Wolbachia (e.g., classic single-copy marker genes for 95% of all Bacteria are not applicable due to genome streamlining, which single-copy genes would be acceptable to determine % complete and % contamination for Wolbachia? What percentage of tRNAs are present in the Wolbachia reference genomes?). It is equally important to try and determine what the %contamination is in each genome – if tools like CheckM (https://github.com/Ecogenomics/CheckM/wiki/Overview) do not work for Wolbachia then another assessment should be implemented. Ultimately, the authors should define their thresholds for selecting genomes to analyze (currently defined as high-quality) separately from the standards used to determine "high-quality" status for a metagenome-assembled genome.

Reviewers' comments:

**Reviewer #1** (Remarks to the Author):

Wolbachia is the most common and arguably one of the most important endosymbionts – and it certainly has received a fair share of attention. Quite surprisingly, for a bacterium with a relatively small genome size, genomic data is still sparse and heavily biased towards a few taxonomic groups. Several times, Wolbachia was discovered as sequencing by-product in studies aiming to sequence its host, but there never has been an attempt at systematically screening *all* short read data from potential hosts (nematodes + arthropods). Scholz et al must be commended for undertaking this stupendous task, and the resulting dataset of >1000 genomes will undoubtedly be very useful to the Wolbachia community. There are two examples given of what kind of questions one can tackle with such a dataset. Probably the most exciting finding (and this may be personal bias) is that Wolbachia evolutionary rates vary a lot between hosts, and that this also varies with host life history traits. This seems very plausible and logical, yet most (even very recent) publications assume that the rate determined by Richardson et al in Drosophila is fixed throughout all Wolbachia/host combinations. Its really nice to have a strong dataset now that demonstrates the variability in Wolbachia evolutionary rates – although I do think that some additional data is necessary to support this claim (see below).
I would very much like to see this paper published, but I want to ask for a few clarifications before I can recommend acceptance of the manuscript – in the hope that this will improve the paper.

Thank you for your overall positive comments. Our answers to your points are as follows.

Major points:

1) Please provide clarification on the number of truly novel genomes that were reconstructed. It seems that many genomes are almost identical to the already published wMel and wRi variants, and others have likely already been described, e.g., in Richardson et al. (https://doi.org/10.1371/journal.pgen.1003129), Turelli et al. (https://doi.org/10.1016/j.cub.2018.02.015), or Choi et al. (https://doi.org/10.1038/nmicrobiol.2016.207). Also, how many of the novel genomes were identical?

Our genome set includes 80 assemblies based on Richardson (2012) samples, and 29 assemblies from Choi (2016). Samples from Turelli (2018) are not included as these were made public beyond our sample download period. We refer to these previous studies by “contains new assemblies of previously described data” in the first sentence of results section 2: “The catalog of newly reconstructed strains expands the *Wolbachia* sampled diversity”. However, to acknowledge that we partly re-assembling genomes from previous work, we changed our title from “Large scale reconstruction..” into “Large scale analysis..”
We would like to underline that in our study we processed all the raw sequencing data in exactly the same way. This enables an unbiased comparative genomics analysis across data from many studies.

Related to this: for novel data from already sequenced genomes (wMel, wRi), wouldn't it have made more sense to simply call variants instead of assembling from scratch? The chance for errors is probably higher when trying to assemble denovo.

We have indeed used a variant calling approach (MUMmer) in order to obtain full-length genome sequences for the hundreds of genomes depicted in figure 4, genomes which are full lengths or nearly full lengths. For the other hosts, we used de-novo assembly in order to allow unbiased identification of novel sequences. We agree with the reviewer that for well-known hosts, reference-based assembly would be a good alternative (as we have done for figure 4). However, here our focus was mainly on identifying a comprehensive set of genomes across a large variety of hosts in order to identify new *Wolbachia* from new hosts. We selected the de-novo approach for all assemblies to extract the genomes in exactly the same way, which allows us an unbiased comparison of the complete genome set, independent from available references and reference genome quality.

2) One problem with reconstructing genomes from such published data is the lack of quality checks – your inference is only as accurate as the metadata associated with the SRA data. I wonder if you could attempt to verify the host species for each of the novel Wolbachia genomes; for example by filtering out the COI gene and blasting that.

This is an interesting point, and in the revision we verified the host species by reconstructing the 18S ribosomal RNA gene sequence. We used the tool RiboTagger. In most cases of successfully 18S reconstruction, we could not find any clear mislabeling of the host species. The host in the majority of our samples could be directly confirmed at the species level. Remaining samples could not be distinguished at species levels, but were confirmed at higher taxonomic levels, such as genus, and subgroup because annotated 18S is missing from the reference database or the 18S fragments used by RiboTagger are identical. In two cases RiboTagger could not confirm the taxonomic assignment of the SRA sample, and we used COI to confirm the exact taxonomy of the source. In few samples we could not extract the rRNA gene sequence, likely because the source data was originally filtered. We modified the main text to update the results on the light of these analyses. Host identification results are added to Supplementary Table 3. Overall, the NCBI host metadata appear to be correct.

3) In your functional analysis, non-CI strains are compared with CI strains, and significantly enriched genes are identified as candidates. This is problematic as there is no phenotypic data for most of the analysed strains. If you make the assumption that all genomes that are very similar to the ones causing CI also cause CI, your argumentation becomes circular: first you group the genomes by similarity and then you identify features that make them similar.

We agree with the reviewer that the lack of phenotypic data might cause some bias and we now report that there is an issue of circularity. However, we would like to point out that for our CI analysis we only selected very similar (nearly identical) *Wolbachia* genomes, e.g. the group of wRi-like genomes consist only of *Wolbachia* genomes that cluster well with the wRi reference genome, same for the wAu-like cluster. Yes, it means we group the genomes by core-sequence similarity, but then we identify the non-core gene content that is different between groups. Moreover, we indeed found that the known CI-genes cifA and cifB are at 100% present in all 234 of our 234 wRi-like (i.e. CI) genomes, and absent in 10 of our 11 wAu-like genomes (considered nonCI, 90.9%), confirming the identity of the wRi genome clusters and the importance of using a statistical

approach in which one single distinct wAu genome does not have a large effect on the result (we double checked that excluding the outlier wAu would not change our list of CI candidate genes). This is exactly the advantage of our statistical approach across many genomes, to be robust against random sequencing and assembling errors, which would not be possible by performing a 1 to 1 reference genome comparison.

The reviewer concern has been presented and discussed in the revised article using the following text:

“For this analysis we selected only very similar genomes and made the assumption that genomes that are very similar to the ones causing (or not causing) CI also cause (or not cause) CI: in the absence of phenotypic information from a large panel of Wolbachia, our assumption was necessary to perform a pangenome analysis which is statistically robust against random sampling. **Although we recon a certain degree of circularity**, we were reassured by the fact that among the 11 candidate genes significantly enriched in CI inducing genomes (Fisher's exact test, $p<10^{-05}$, Bonferroni corrected, Supplementary Table 4), five of them, including *cifA* and *cifB* were previously identified by LePage et al. 2017.”

Also, I think there may be a systematic biased introduced in testing for enrichment by including this many wMel and wRi variants which are essentially identical in gene content.

To avoid a potential bias due to the many variants, we excluded 79 genomes that show an identical core sequence to other genomes. This affected slightly the p-values, but does not change the list of our CI candidate genes. The larger number of genomes itself in CI vs non-CI does not cause problems by using Fisher test statistics. It’s the fraction/percentage of a gene present in one group vs. another, independent from group size.

Further, you should compare the genes that you have identified with the candidates identified through comparative genomics in LePage et al (https://doi.org/10.1038/nature21391), there may be some overlap.

The two main candidates (cifA and cifB) do indeed overlap with LePage data as already indicated in the text. We have followed the reviewer suggestions and further compared it with the supplementary tables S1 and S2 of LePage. Two more of our genes are present in the CI-gene-table LePage:S1, and another gene is confirmed in the wAu-absence table LePage:S2.
To allow an easier comparison of identified CI candidate genes, we added the wMel-reference gene identifiers as used by LePage into our Supplementary Table S4. Compared to the long lists presented by LePage (161 genes in table:S1 and 60 genes in table:S2) based on comparing individual reference genomes, our statistical approach identifies a compact list of only 11 CI-candidates of highest relevance as these genes fulfill the stronger criteria of being significantly enriched across many genomes. This new information is provided in the revised article.

Finally, if you find phage genes significantly enriched, this is not that surprising, as the CI loci are located in a prophage region.

Thank you for pointing this out, we added it to the text. The complete paragraph reads now:

“Our pangenome analysis reveals six *{not nine as before}* additional genes with functional annotations related to, among others, riboflavin, benzoate, and a bacteriophage. While significantly enriched phage genes might not be surprising, as the CI loci are located in a prophage region, other candidates may play a role in CI biology and should be investigated further experimentally.”

4) I think to demonstrate that Wolbachia evolution is indeed as heterogenenous as claimed, one might also have to look at nuclear data. If you only compare rates of mitochondria and Wolbachia, it could also be that it’s the mitochondria that actually evolve at very different speeds between taxa (which is not totally unreasonable to assume), whereas the Wolbachia might evolve equally fast. It’s a very good dataset to test this– you should try and extract a couple of housekeeping genes that are present in all of the hosts and determine their evolutionary rates.

This is an interesting point (which has been also suggested by colleagues) and we were initially planning it. However, there is a difference in the genetic inheritance between mitochondrial and nuclear genomes which makes this type of analysis not applicable for intraspecific (population scaled) samples. While mitochondria and *Wolbachia* are uniparentally (maternally as far as we understand them) inherited, nuclear genes follow a classic mendelian inheritance, with alleles coming from both mother and father. This generates a condition of coalescence which impedes to follow a tree like structure for nuclear genes inheritance. Furthermore, because of diploidy for every sample we would have 2 alleles for each of the genes) with the impossibility of defining which is one. The use of housekeeping nuclear genes would be ok for comparing *Wolbachia* between different host species because in this case we can assume that allele variants have been fixed during speciation (even though there may be some Incomplete lineage sorting). The same approach would not work for intra-specific individuals. Now this is briefly explained in the article to avoid readers to ask themselves the same question that we (and the reviewer) ask. However, the reviewer has raised the interesting point that is the “mitochondria that actually evolve at very different speeds”. This is possible, although it may not explain a twofold difference. We now acknowledge this possibility in the text: “We did not assemble host nuclear data because while mitochondria and *Wolbachia* are uniparentally inherited, nuclear genes follow a Mendelian inheritance which impedes building a genealogical (tree-like) structure[33].”

5) Please upload the novel genomes to a permanently accessible database, such as Datadryad – its not that expensive (only 2% of the Nature Communications APC) and will ensure long term access for the community.

Now, we submitted our genomes to the EMBL-EBI European Nucleotide Archive (ENA) referenced in the Data Availability statement (Study-ID: PRJEB35167). ENA genome accession IDs are added to supplementary assembly table S3.

Minor points & suggestions, hopefully constructive:

Title
Suggest to delete ‘Large scale’

Considering that we have screened the whole SRA repository and retrieved hundreds of assemblies we would like to keep the term “large scale”.

Line 48
There may be only 43 reference genomes, but if you define genomes as you define them in the title of your manuscript, the number is substantially larger (see major comment 1).

In our work we define as reference genomes those *Wolbachia* genomes that are officially labelled and recognized by NCBI as *Wolbachia* genomes. These genomes are arguably the first reference for genomic studies of newly reconstructed genomes.

Line 57
Suggest to change 'because of no phylogenetic signal' to 'due to lack of phylogenetic signal'

We replaced it as suggested.

Figure 1
This should be 2 separate Figures, 2b is barely readable.

We do not completely understand whether the reviewer refers to figure 1 or 2. Both figures are indeed very information-rich. But since the figures could be printed as vertical page, we would like to let the final decision to the print/editorial office.

Line 111
I couldn't find information on how the host genome size was determined. Are all estimates from the literature?

Yes, for well-curated genomes such as *Drosophila* we used the direct genome estimate, otherwise we have used an extensive database of genome size based on C-values http://www.genomesize.com/. This is now mentioned in the caption of figure 1. The C-values and the sequenced genome size were very similar.

Line 168–169
These genomes are published: https://doi.org/10.1101/461574 and http://dx.doi.org/10.1101/551036

Thank you. We included these references into the revised version of our manuscript.

Line 177
Various bees -> 2 bee species (according to your Table S3)

You are right. We replaced "*various bees*" with "*two bee species*".

Line 178
Thetranycus urticae -> Tetranychus urticae

This typo has been fixed.

Line 195–198
These are examples of why it would be good to verify the host species with COI or similar. For example, Holcocephala fusca is a robber fly and thus predatory. Depending on the DNA prep it is very possible that this Wolbachia strain is from a prey individual, rather than from the robber fly. Also, without demonstration that there is no other potential host species in the sequencing data of Caenorhabditis remanei, I find it very difficult to believe that Wolbachia is actually present in this species. To my knowledge, this would be the first Wolbachia strain detected in the Rhabditidae – all other nematode-Wolbachia associations are limited to the filarial nematodes.

Using RiboTagger, we could confirm the host *Holcocephala fusca* (SRR1738186) at the genus level, being similar to the 18S reference of *Holcocephala abdominalis* (the 18S of *H. fusca* is not yet deposited in GenBank). We further check for signs of contamination by blasting the SRR1738186 using COI of Drosophila melanogaster as query and found few reads with 95% similarity: blasting of these reads against the nucleotide collection returned as best hits various hoverflies (*Sphaerophoria* sp. and *Eristalis* sp.). The intuition of reviewer was correct, and it is possible (but unlikely given the very few non *Holcochephala* 18S fragments) that the wMel-like *Wolbachia* is from one of those prey (but not from a Drosophila) of the robber fly. We added text to themanuscirpt: "Although 18S screening confirms this SRA as *Holcocephala*, we found some reads with high similarity to the cytochrome oxidase subunit I (COI) of hoverlies. We therefore cannot exclude that our reconstructed *Wolbachia* genome is not from the robber fly itself, but from an hoverfly prey. Because we can exclude a contamination from a *Drosophila* prey (suppl. Information), this strain indicates a ..."

For *Caenorhabditis remanei* (SRR275642), we could not reconstruct any 18S sequence using RiboTagger due to low sequencing depth, and indeed this sample is considered to be of lower quality, present only in the gene-content tree (Fig. 2b), and not part of our genome set in the core sequence tree (Fig. 2a). We manually blasted SRR275642 using *Caenorhabditis remanei* Cytochrome Oxidase subunit I (COI) and found reads covering the whole gene with 99-100% identity, confirming the exact source of this sample. However, in order to exclude contaminants we further blasted using *Drosophila mauritiana* COI (AF200831.1) because the putative new genome for *Caenorhabditis remanei* has 98% identity to wNo of *Drosophila mauritiana* in our Fig. 2b. Indeed, we found reads covering a portion (not all) of the gene with 98-100% identity, indicating a contamination from a *Drosophila mauritiana* or from another closely related *Drosophila*. We modified text adding: "We further identified a dubious new strain in the nematode *Caenorhabditis remanei* with a 98.04% identity in gene content with wNo of *D. mauritiana* (**Fig. 2b**). While we could not verify the latter using 18S, we found 100% similarity with the annotated COI of *C. remanei.* We found however some reads covering a portion of *D. mauritiana* COI: since no *Wolbachia* has ever been found in the *Caenorhabditis* genus, it is possible that this *Wolbachia* comes from a contamination. Indeed the genome from this sample did not pass our quality control and was considered only for our gene-content tree (Fig. 2b and not Fig. 2a). These two cases highlight the difficulty in determining the exact source of some samples when reconstructing endosymbiont genomes using a metagenomic approach."

Line 205–206
I can't follow this line of argumentation: most Wolbachia from Hymenoptera in your supergroup A tree are not from parasitic lineages.

The reviewer is right. We have fixed the text to exclude a role of parasitoids as it is not supported by our sampling. The paraphyletic Hymenoptera pattern we observe is however quite interesting and we think it deserves mentioning.

Line 222–224
This is not surprising, as most of the genomes from these groups would belong to highly adapted strains with degraded genomes.

Thanks. We added this comment to the text.

Line 370
Please clarify “arthropod and nematode host related keywords”

We have briefly explained in the main text, and we now provide the full list of keywords in supplementary information.

Line 415ff
Please clarify: does this mean all contigs of one metagenomic assembly would have to match *the same* or *any* Wolbachia reference?

It should mean “*any*”, we added it to the text.

Line 428ff
Is number of contigs not considered important to call an assembly “high quality”?

Our quality criterion is primarily focused on the presence of genes and on the polymorphic rate (chimeric quality) of our genomes in order to perform a robust gene-functional analysis. Yes, fragmentation is also important and we report the N50 quality measure in table S3. We have not included fragmentation into our quality threshold criteria as we already exclude short contigs <1000nt to be part of the genome, and hence indirectly limit a too extreme contig fragmentation.

Line 437ff
That’s a very cool approach to determine if multiple Wolbachia strains are present!

Thanks!

Line 474
“pident” -> “percent identity”

Thanks, we replaced it.

Line 476ff
In terms of alignment accuracy, it might make sense to align the loci separately and then concatenate. Maybe also exclude recombining loci?

Yes, correct (concatenating after alignment). We made and error describing it in the text. We corrected the methods section.

Supplementary information

Line 24ff
Please avoid using “basal” to describe the position of taxa in trees.

We replaced “*basal*” by “*closer to the root*”

Line 128
“incongrences” -> “incongruencies”
“Cimex lecturalis” -> “Cimex lectularius”

We have fixed these typos.

There are 3 metagenomic samples in the dataset (2x "Apoidea", 1x "Insecta") – I wonder if it makes sense to include these here, as a host cannot be assigned.

We added the most likely host, based on 18S rRNA reconstruction (provided for all samples in table S3). Insecta is most likely a moth. The two Apoidea samples are from bees, potentially Apis mellifera. We added more specific host details to the tree in figure 2.

**Reviewer #2** (Remarks to the Author):

Scholz et al., review

Wolbachia is an important intracellular symbiont that has - and continues to impact insect populations and evolution. Since the sequencing of the first Wolbachia genome in 2004, there have been many efforts to increase sampling of Wolbachia across the genus (clades) and from different host species (orders of insects, other arthropods, nematodes, etc). Here, Scholz et al mine publicly available sequences from the SRA to identify possible Wolbachia sequences/reads based on mapping reads to available Wolbachia reference genomes (43 total currently). Their pipeline for identification of Wolbachia reads is straightforward and this approach (mapping to existing genomes) has been used by others.

Thanks for the summary. We would just like to point out that, in order to allow identification of novel sequences, we are using de-novo assembly, not mapping against reference genomes.

The authors claim to identify over 1,000 new Wolbachia genomes using this method - indeed, that would be a triumph and could provide a lot of interesting information about Wolbachia evolution and horizontal transfer between species.

My main concern is the lack of caution used with regards to potential contamination from other bacteria (the microbiome) and from host lateral insertions (Wolbachia genomic fragments inserted into host nuclear genomes).

We agree that it is important to address the issue of potential contamination. In order to identify potential contamination from other bacteria, we mapped all our 1006 genomes against 99,154 reference genomes from NCBI-GenBank. Based on a BLAST default settings and threshold of >97% identity over > 10k nucleotides, we identified only one hit of a *Drosophila melanogaster* related *Wolbachia* strain (SRR1183652) that includes sequences that are similar to *Acetobacter* related species. We marked this *Wolbachia* strain as of low quality, reducing our identified genomes to 1005. Also a more sensitive BLAST search (-task blastn -word_size 11) did not reveal any additional contamination hit.
As for the host contamination, because we have initially mapped against VERIFIED Wolbachia genomes (and later in the pipeline we de-novo assembled), then it is unlikely that we have host genomes inside the *Wolbachia* genome. However, the reviewer is right that there may be problems in the cases of *Wolbachia* fragments inserted into host genomes: for this reason we have manually checked for such cases as explained in the next point.

There are some major flaws, based on incorrect assumptions, in these data and their analyses. First, Wolbachia are well known to integrate into their host genomes - so called “lateral gene transfer” or LGT. Many of the reads that the authors identify across insect species could come from these LGTs and there is no validation of their data to account for robustness against LGTs. The authors set a threshold of 50% core genes for inclusion of these genomes but frankly, this is much too lenient. We know at least two cases where entire Wolbachia genomes are integrated into the host nuclear genome.

The threshold of 50% core genes refers to the global core gene set across all supergroups. Core genes are not necessarily present in all supergroups. When looking at each non-reference *Wolbachia* genome we used in our analysis, we found that they have on average 88% of their supergroup-specific core genes.

We acknowledge that our pipeline may be sensitive to possible nuclear integrations inserted in the genomes. This however, according to reviewer and to the literature, should be a problem only for two hosts, *Callosobruchus chinensis* and *D. ananassae*. We therefore manually checked our core-genome data (the one used for our main tree of figure 2) for these hosts. According to Choi et al. (2015, GBE) integrated *Wolbachia* are characterised by an excess of non synonymous mutations as well as stop codons and frameshifts because of relaxed selection on the integrated *Wolbachia* compared to non-integrated one. We first realigned the core genome for these two hosts in order to restore the codon frame of genes (the core genome is mainly composed of conserved Wolbachia coding genes), we then look for intergenic stop codons, frameshifts, as well as for region poorly aligned. We did not find any internal stop codon nor disruptive insertions/deletions, except for few poorly aligned fragments, which we have blasted and all look genuinely as *Wolbachia*:

- In one of the two *Callosobruchus Wolbachia* (SRR949786), we found a poorly aligned sequence at position 22673 of the core genome alignment (position 4588 of Callosobruchus alignment) 1638 nucleotide long. This regions however does blast with 99.5% identity (and zero gaps) against well annotated *Wolbachia* genomes such as wPip and wAlbB.
- In *D. ananassae* in SRS2127163 we found a 1757 nt fragment at position 19245 of the core genome) which however blast with 100% similarity to *Drosophila ananassae* strain W2.1 chromosome (wAna, cp042094.1) and with wRi (CP001391.1).

- In *D. ananassae* SRS2127151-2127152-2127153 we found a 489 nt fragment at position 6132 which blast 100% with *Drosophila ananassae* strain W2.1 chromosome (wAna, cp042094.1) and wRi (CP001391.1).
- In D. ananassae SRS2126857-2126916-21235641-2127154 at position 36570 a fragment of 783 nt blasting 100% with *Drosophila ananassae* strain W2.1 chromosome (wAna, cp042094.1) and wRi (CP001391.1).
- In D. ananassae SRS2126857-2126916-21235641-2127154-2135644-2135642 at position 181210 of 993 nt blasting 100% with *Drosophila ananassae* strain W2.1 chromosome (wAna, cp042094.1) and wMel_ZH26 (CP042445.1)

The fact that all these fragments blast with perfect or nearly perfect similarity with well-curated reference genomes such as wRi, wMel and wPip, and that we did not found any present insertion/deletion when compared to them reassured us that they are genuinely *Wolbachia* fragments and not host integrated fragments. These new analyses are now in the supplementary information.

Furthermore, we also inspected our MUMmer alignment of *D. ananassae Wolbachia*, and did not find any evidence of wAnaINT: because according to Choi et al. 2015 (table 2 and table 3 therein) wAnaINT accumulate 20 times more mutations than wAnaINF, we would expect an excess of mutations (therefore long branches in a phylogeny) in samples contaminated by wAnaINT fragments: the branch length of all samples is instead homogenous (similar to each other) according to a RAxML analysis of the dataset.

We however agree with reviewer that integrated fragments may be an issue for our wAna genomes and we have modified text to acknowledge this: "For *D. ananassae* we excluded bias from wAnaINTs (*Wolbachia* genomes integrated in host genomes) data accidentally included in our assemblies: first of all, we could not find fragments attributable to integrated *Wolbachia* (see **Supplementary Information**); second, as integrated genomes evolve neutrally, or almost neutrally[29], they cannot produce a phylogenetic signal incompatible with that of the host genome. Because of its peculiar integrating genome biology, we nonetheless advocate caution in the interpretation of our results for wAna. "

Also, they do not account for potential bacterial reads that map to the conglomerate Wolbachia assemblies just due to homology - this is indeed a major issue that we have seen many times in resequencing Wolbachia genomes. Similarly, the number of polymorphic reads they allow in their assembly is very high for Wolbachia infection. I contest the use of "high quality" throughout — a "high quality" genome would have a less fragmented assembly.

We followed the reviewer's advice and no longer use the term "high quality" for our selected genomes that fulfilled our quality requirements. Additionally, as described above, the newly performed BLAST mapping against nearly 100,000 reference genome shows a potential contamination issue in only a single assembly.

The fact that the authors could identify multiple paralogs of Wolbachia "core genes" by Blast within their "high quality" genomes should have been a red flag.

Also, there seems to be no curation of the SRA content before they run their assemblies - these datasets could come from pools of animals or environments, making downstream interpretations

really difficult. How can you say that there is a 5% prevalence if the samples are pools of 100 flies, for example?

Even though we have screened few pool-seq samples, we excluded pool-seq samples from our screen when clearly labelled in the NCBI metadata. Most of our screened samples can therefore be considered as not being pool-seq. Samples identified later as pool-seq were marked in our phylogenetic tree (Fig.2). Pools-seq samples usually look like having multiple *Wolbachia* infections and hence are supposed to show a high polymorphic rate or large genome sizes which results into assemblies considered as of low quality. Most pool-seq samples are even of very low *Wolbachia* sequencing depth, not suitable for assembly, and hence only present at lower quality in the gene-present-absence tree, and are not part of our *Wolbachia* genome set. But we agree with the reviewer, and added to the text that “The reported prevalence levels might be affected by the sometimes limited metadata quality of the NCBI repository and our sampling strategy.”

Finally, with regards to the bioinformatics, there is absolutely no benchmarking or validation on datasets KNOWN to be infected or uninfected. That would’ve provided better confidence. For example, the D. ananassae assembly has an integrated Wolbachia genome - how does their pipeline perform in the face of that?

Our screen included also source samples from *Wolbachia* reference genomes (see full phylogenetic tree, Supplementary File 1). If original source samples are available (not available for wAna, but for wRec, wDi, and wSuzi), we see that our extracted assemblies are nearly identical to the reference genomes (>99.9% genetic identity), confirming reproducibility and quality.

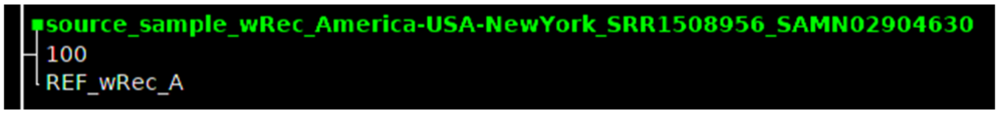


Unfortunately, we don’t have the source sample of the wAna reference genome, but the wAna reference is well embedded in the cluster of our *D. simulans* and *D. ananassae* assemblies, confirming a high sequence similarity. And, as additional confirmation, we manually screened our wAna assemblies for potential host integrated sequences as described above.
We report the three examples in the Methods: “we could confirm the quality of our assemblies by re-assembling Wolbachia reference genomes from available source samples. Based on a core sequence length of more than 300,000 nt, we found 99.99% genetic identity between our assemblies (SRR183690, SRR1508956) and the original reference genomes of wDi and wRec, and even 100% core identity between our assembly (ERR188908) and the reference strain wSuzi.”

More minor comments:

Line 124: “reduced genomes” or “shorter genomes relative to the other strains”
- We replaced “short genomes” with “reduced genome length”.

**Reviewer #3** (Remarks to the Author):

Overall, I think the manuscript is well reasoned and written. The effort undertaken by the authors is

large and the results seem equally impactful to the community at-large. I appreciate the succinct language and high quality of the writing – the manuscript was enjoyable to read. I have a few small comments/questions for the authors and then 2 larger concerns (one which should be easy enough for the authors to adjust).

We thank the reviewer for the positive comments. The manuscript has been revised as described below.

Small concerns/typos/questions:
Ln 196-197. What method was used to determine the % nucleotide identity here? I could not find it in the methodology.

Genetic identity of *Holcocephala fusca* was calculated by counting the number of base mismatches along the core-gene-alignment, ignoring gaps. The gene content identity of *Caenorhabditis remanei* was calculated by comparing the co-presence and co-absence of genes with presence/absence mismatches along the PanPhlAn pangene profiles. We modified the text to make it more clear that *Holcocephala fusca* % identity is based on the "core" sequence.

Ln 380. I was unclear as to what "pre-selected" referenced? I believe it's the 1,801 samples that had quantifiable Wolbachia signature?

Yes, correct. We modified the text to make it more clear.

Ln 458. How were representative selected?
Based on the clustering described in the next sentences. We added to the text: "as follows:" and clustering of "all" genomes to make it more clear.

Ln 471. pident is an abbreviation and should be explained for readers

We replaced "pident" with "percent identity", as suggested by reviewer 1.

Ln 504. In this instance does "sequences" mean "genomes"? If so, please change. If not, please clarify.

Yes, genomes is correct. We replaced "sequences" with "genomes".

MUMmer assemblies. I understand the need to implement an alternative method when the datasets get large, so the switch to a MASH based approach for strains infecting D. melanogaster and wRi strains is logical. I just wonder the impact this has on the output presented in Figure 4. Is there any way of determining these results using the same methodology for all? Could the MASH approach be used for the smaller datasets and then any findings could more definitively linked to some underlying biological principle as opposed to possible influence of methodology?

We changed the title of the section from "MUMmer assemblies" to "MUMmer alignments" because this reflects more closely what was actually done. We also would like to point out that the pruning of the *D. melanogaster* and of *wRi* strains *D. simulans* was done only to reduce the number of sequences for downstream analysis. The filtering discarded some of the sequences when almost identical sequences were present. From a technical point of view, the reason for this additional

step was that aligning a large number of incomplete genomes can lead to a small core genome just for the stochastic distribution of missing regions. A sentence was added in the text to clarify this point.

Roary for pangenomes. I'm not personally not up on the current accepted understanding of Wolbachia phylogeny, but in reviewing Roary it came to my attention that there are several warnings in the FAQ against using this tool for organisms outside of the same species. Now the Fig. 2 tree clearly illustrates that Wolbachia genomes from D. simulans could be considered strains of the same species, but my interpretation of the tree would be that the supergroups likely represent different "species" of Wolbachia. Possibly? The Roary settings used by the authors is 80% nucleotide identity. The Roary FAQ explicitly states that 70% would be too loose to be meaningful and recommends the lowest value of 90% identity for "diverse species". I wonder on what the results were for the authors when using the default and 90% cutoffs? And if the authors considered adjusting the mcl inflation value to influence clustering results? Determining where the species cutoff is for these groups may be aided (and would place these genomes in the context of other elements of the bacterial tree of life) by using the GTDB-Tk tool (https://github.com/Ecogenomics/GTDBTk).

We needed to lower the percent identity threshold to 80% in order to identify a reasonable large number of core genes that are present across all the different supergroups (present in at least 1 genomes of the selected 5 representative genomes per supergroup). A very stringent similarity threshold of 95% would result in only 6 core genes and a very high number of pangenome genes. Also by using 90% we would still get only 45 core genes. With the selected 80% we get 316 core genes, which from our experience is a good balance between getting a large number of core genes from a not too large number of pan-genes, and still controlling to have functional distinct gene-families. We agree, using a very low threshold, such as 70% could lead to an incorrect clustering of functional distinct genes into a single gene-family. To illustrate the similarity threshold effect we re-run the Roary clustering using different threshold from 70% to 95%. The resulting number of core and pangenome genes are shown in the following table:

| BLAST pident th | 70% | 75% | **80%** | 85% | 90% | 95% |
|---|---|---|---|---|---|---|
| Pan-genes | 6810 | 7105 | **7442** | 8224 | 9354 | 11053 |
| Core genes | 508 | 428 | **316** | 170 | 45 | 6 |

Concerns:
(1) Storage of the genome results in a detected Google site is one way of making the data public, but I suggest that authors instead store the data in official repository that includes version tracking, DOIs, and robust legacy protocols, such as figshare or Dryad. While custom websites achieve this goal at bare minimum level, ensuring that the data is preserved in perpetuality should a website break or webmaster level, etc. should be main priority of making the data available, public, and reusable. I would recommend the authors review the FAIR practices for data availability (https://www.nature.com/articles/sdata201618).

As suggested also by Reviewer #1, we submitted our genomes to a permanently accessible repository: the European Nucleotide Archive, EBI-ENA ID: PRJEB35167.

(2) This concern has to do with how the new Wolbachia genomes fit in to the current language and accepted practices for metagenome-assembled genomes (MAGs). It is clear that the “genomes” for this manuscript are in fact, as the polymorphic data presented in Supplemental Table 3 confirms, MAGs and consist of a population-level genome-equivalents. The authors should make it clear to the readers that these genomes are not pure isolate genomes and therefore should not be treated as such.

We definitely agree on this point. We have now discussed this already in the intro:

> “Although MAGs cannot reach the quality of isolate genome sequencing which is unavailable for intracellular parasites, this large catalog of *Wolbachia* MAGs allowed us to infer robust phylogenies, identify new variants, build host population level datasets, and ultimately clarify some open questions concerning *Wolbachia* evolution.”

And discussed in the results

> “We metagenomically assembled 1,166 genomes – called metagenome-assembled genomes, MAGs - from our 1,793 positive samples (see **Methods**),”

And

> “We defined MAGs quality based on four main criteria (see **Methods** and **Fig. 1b**) as a strict control to retain a total of 1,005 *Wolbachia* MAGs, which we have used to infer a whole-genome phylogeny based on the alignment of 316 core genes (**Fig. 2a**). Although MAGs are inherently less accurate than genomes obtained by isolate sequencing […]”

To further address this, the authors need to put their genomes and definition of “high-quality” in the same context as other MAG datasets and the practices detailed by Bowers et al. (https://www.nature.com/articles/nbt.3893). Wolbachia may be a great edge case as to why these standards may not be appropriate, but these guidelines should be adhered to, if possible, and acknowledge and explained why if they cannot be. If not possible, I would recommend that the authors develop similar standards to Bowers et al. that are logical for Wolbachia (e.g., classic single-copy marker genes for 95% of all Bacteria are not applicable due to genome streamlining, which single-copy genes would be acceptable to determine % complete and % contamination for Wolbachia? What percentage of tRNAs are present in the Wolbachia reference genomes?). It is equally important to try and determine what the %contamination is in each genome – if tools like CheckM (https://github.com/Ecogenomics/CheckM/wiki/Overview) do not work for Wolbachia then another assessment should be implemented.

Ultimately, the authors should define their thresholds for selecting genomes to analyze (currently defined as high-quality) separately from the standards used to determine “high-quality” status for a metagenome-assembled genome

We removed the term “high-quality” to avoid confusion with the MIMAG quality standard definitions. And we checked our assembled for potential contamination, see comment to reviewer #2 and modified the Methods as follows: “To control for potential sequence contamination, we mapped all our passed QC assemblies against nearly 100,000 bacteria reference genomes from NCBI GenBank. Using a BLAST threshold of >97% identity over a minimum alignment length of 10,000

nucleotides, we identified only one *Drosophila melanogaster* related *Wolbachia* strain (SRR1183652) that includes sequences that are similar to *Acetobacter* related species."

Reviewers' comments:

Reviewer #1 (Remarks to the Author):

In the revised version of their manuscript, Scholz and coworkers have carefully and comprehensively addressed most of the issues I identified with the first version of the manuscript. I want to commend the authors for a very important piece of work that will prove useful for the Wolbachia community. I have one major point left that I would like to see addressed, and I am only insisting because I genuinely belief it will make the paper a lot stronger:

In you rebuttal, you argue that it is not sensible to look at nuclear data because of incomplete lineage sorting. My response would be that you don't really need to reconstruct trees to show that Wolbachia genuinely evolves at very different speeds between host lineages. Could you not simply extract a nuclear locus (you already did this for 18S rRNA!) with very low expected heterozygosity levels from all individuals of a host and estimate average genetic divergence for the whole population on that locus? This number can then be used to normalize mitochondrial rates of evolution, which in turn would then be more useful to determine Wolbachia evolutionary rates. In other words, instead of looking at Wolbachia / mitochondrial rates you should look at Wolbachia / mitochondrial / nuclear rates. If mitochondrial / nuclear rates are ~identical across different hosts, your estimated differences of Wolbachia evolutionary rates are more likely to be real. It is a very simple calculation (1 rate for each host species), and the estimates will likely be crude, but it will also make your claims much more robust.

Reviewer #2 (Remarks to the Author):

Although the authors have written a substantial amount, the authors have not adequately responded to one of my prior concerns. Importantly, because they have not checked their SRA datasets to ensure that they are true Wolbachia genomes and not LGTs, I question their presentation of their results as MAGs and their conclusion regarding evolutionary rate variability. A simple solution would be for the authors to use the approach in the wWb manuscript (https://academic.oup.com/femspd/article/75/9/ftx115/4584485) at least for those lineages that are 1) new to the field and/or 2) show evidence of evolutionary rates. I would then feel more confident in their results.

Reviewer #3 (Remarks to the Author):

The authors have made significant progress in advancing the state of the manuscript. I still have a few concerns that were not satisfactorily addressed in these revisions but should not prove problematic to the authors.

In the response to Reviewer #2 the authors state: "We followed the reviewer's advice and no longer use the term "high quality" for our selected genomes that fulfilled our quality requirements." Yet, the term high-quality still continues to be appear in parts of the manuscript – mainly Figure 1B – high-quality vs. core-quality. This continues as issue that was articulated by Reviewer #2, but I had (and am still having) is the divide between mapping and de novo methods. In my understanding: "Mapping" is used to determine which samples have Wolbachia infection. Then de novo assembly occurs. And then "genome bins" are formed by "mapping" contigs against the 43 reference genomes. Then there is metric to determine a positive match. Somewhere, between Ln 445-499 there should be a clear definition of (formerly) high-quality vs core-quality, as the significance of these meanings is still unclear.

I am in agreement with Reviewer #2 that this method leaves open a wide gap for co-infection,

chimeric assemblies to persist in the data (which is okay for this type of dataset). This is supported by both the polymorphic rate (as reported by the authors) and the need in Ln 521-522 for the authors to select one representative when there are multiple copies of a core gene. The authors have not identified these core genes as single copy, but in practice many of the gene seem to be and a threshold should be applied that the core genes are believed to be single copy in the genome. The co-occurrence of core genes in a single MAG is generally referred to as “strain heterogeneity” in tools like CheckM and represent incorporation of multiple populations into a single MAG. Instead of selecting a representative of these core genes, which if incongruent with the other genes in the phylogenomic analysis will lead to longer branch lengths, the authors should exclude duplicate core genes in the alignment. I think the inclusion of polymorphic rate as a measure of population diversity is useful, overall, the authors could add emphasis of the role the population plays in the final dataset.

I do not think that the amended methodology Ln 486-498 is sufficient to satisfy this concern.
(1) Why only consider contigs >10kb, when contigs >1kb are included in the MAGs and shorter contigs may be the most problematic in terms of contamination?
(2) The threshold of 97% nucleotide identity over 10kb is too strict for the identification of genetic material for potentially novel species (95% NAID) or genera (90% NAID). The authors should have a much looser cutoff here, with the goal of removing any possible contamination, airing on the side of false positives to ensure quality. Any %identity match at 10kb above 85%NAID to an organism other than Wolbachia would be problematic and should be removed. An alternative method to this BLAST approach would be to compare all sufficiently large contigs (>10kb) against the reference database using FastANI and removing any non-Wolbachia matches.

Typos and clarifications:
Throughout: Bowtie2 is reference multiple times, each with a different spelling (e.g., BowTie2, bowtie2, etc.). Please unify with the correct spelling.
Ln 210. “hoverlies” should be “hoverflies”
Ln 213. The authors should still indicate their uncertainty by changing “this strain indicates a” to “this strain likely indicates a”
Ln 258. “recon” should be “recognize”
Ln 446. “the 1220 Wolbachia-positive samples”. Should be clarified as stated in Ln 415-417, these are specifically samples with >4X coverage.

Reviewers' comments:

**Reviewer #1** (Remarks to the Author):

In the revised version of their manuscript, Scholz and coworkers have carefully and comprehensively addressed most of the issues I identified with the first version of the manuscript. I want to commend the authors for a very important piece of work that will prove useful for the Wolbachia community. I have one major point left that I would like to see addressed, and I am only insisting because I genuinely believe it will make the paper a lot stronger:

In your rebuttal, you argue that it is not sensible to look at nuclear data because of incomplete lineage sorting. My response would be that you don't really need to reconstruct trees to show that Wolbachia genuinely evolves at very different speeds between host lineages. **Could you not simply extract a nuclear locus (you already did this for 18S rRNA!) with very low expected heterozygosity levels from all individuals of a host and estimate average genetic divergence for the whole population on that locus?** This number can then be used to normalize mitochondrial rates of evolution, which in turn would then be more useful to determine Wolbachia evolutionary rates. In other words, instead of looking at Wolbachia / mitochondrial rates you should look at Wolbachia / mitochondrial / nuclear rates. If mitochondrial / nuclear rates are ~identical across different hosts, your estimated differences of Wolbachia evolutionary rates are more likely to be real. It is a very simple calculation (1 rate for each host species), and the estimates will likely be crude, but it will also make your claims much more robust.

Thanks to the reviewer for insisting. We followed the suggestion and examined carefully the idea of using a nuclear marker and concluded that 18S rRNA is likely the best candidate. We previously used RiboTagger to extract the hypervariable V4 region in order to confirm the host's source (Supplementary Table S3). RiboTagger, however, is limited in extracting short sequence length. We therefore additionally mapped all our reads against full length 18S host reference sequences and reconstructed a full set of 18S alignments for each of the 11 host populations of Figure 4.

We then compared the average genetic distances of their *Wolbachia* with that of the 18S and the mtDNA. As expected from the co-phylogenies of Figure 4, there is no correlation between *Wolbachia* and mtDNA divergences (new plot of Figure 4b). Instead, we find a significant correlation between *Wolbachia* and 18S divergence time. This is very interesting as it indicates that *Wolbachia* indeed follow the molecular clock of the nuclear hosts. Mitochondria, in contrast, are characterised by a rate that departs, apart from a few cases, from both the *Wolbachia* and the nuclear data.

Our explanation is that the mitochondrial genomes of 4-5 species are characterised by a peculiar genetics. Indeed, when we exclude these species, we recover a good correlation, and notably with almost the same slope of the Nuc-Wolb correlation. The problem seems to be related to the mtDNA of some hosts. This finding deserves future corroborations in particular by analysing large chunks of host nuclear genome, something complicated by the

high variability of animal genomes and the unease of selecting markers that are under similar (as neutral as possible) evolutionary pressure between the various host populations. We believe that this goes beyond the current scope of this article, but we are indeed planning studies in this direction. Still we believe that the evidence presented in our new Figures 4b and 4c are exciting and deserves to be put to the attention of the community. Indeed the new results reinforce our point that we need to be extremely careful in using mtDNA data to calibrate the clock of *Wolbachia*.

**Reviewer #2** (Remarks to the Author):

Although the authors have written a substantial amount, the authors have not adequately responded to one of my prior concerns. Importantly, because they have not checked their SRA datasets to ensure that they are true Wolbachia genomes and not LGTs, I question their presentation of their results as MAGs and their conclusion regarding evolutionary rate variability. **A simple solution would be for the authors to use the approach in the wWb manuscript (https://academic.oup.com/femspd/article/75/9/ftx115/4584485) at least for those lineages that are 1) new to the field and/or 2) show evidence of evolutionary rates.** I would then feel more confident in their results.

We have included the suggested LGT measures. We screened all our MAGs for low-confidence regions, following the procedure of Chung (2017). Supported by both criteria, high sequence depth and variation together, we found only one MAG (ERR068352, *Brugia pahangi*) with potentially contaminated regions higher than 1% according to both high coverage and high sequence variation, and 7 MAGs with further high coverage variation higher than 1%. The number of MAGs with high sequence variation higher than 1% were 169: this is indicative of some genetic heterogeneity in the *Wolbachia* within a single host and is in agreement with the level of polymorphic rate we have previously reported in our Suppl table 3. Not surprisingly, the MAG with the highest sequence variation (5.84%) was found in a Pool-seq sample of *D. simulans* (SRR2036958) which was already considered as of low quality by our other QC criteria and excluded from the core tree.

Since LGT shall generate a significant increase in coverage variation, we conclude that only the *Brugia pahangi* and the other 7 MAGs are potentially misleading due to possible LGT. 5 of these MAGs are from nematode *Onchocerca volvulus* hosts, and one from the bee *Lasioglossum albipes*. <u>None of these MAGs are involved in any of the putative within host transfers (dotted line of Figure 4) or exhibit any peculiar rate of evolution in accordance with Figure 2.</u> The new low-confidence quality scores of all MAGs are added to Supplementary Table 3 and are discussed in the main text:

*"We detected various instances of increased sequence variations at 1-6% length, but only eight assemblies with increased coverage regions at 1-3% length mostly in nematode hosts. (Supplementary Table 3). Because Wolbachia-host lateral gene transfers (LGTs) shall generate a significant increase in coverage variation, we conclude that only eight of our MAGs are potentially affected by misleading signal related to LGT. None of these MAGs are involved in any of the putative within host transfers (dotted line of Figure 4) or exhibit any peculiar rate of evolution in Figure 2. However, because we found a certain level of genetic*

*heterogeneity, we cannot completely exclude confounding factors from multiple Wolbachia infections in some of the MAGs.”*

And in the Methods section:
“*Additionally, we screened our assemblies for low-confidence regions defined by unexpected higher coverage and higher sequence variation compared to average, following the procedure of Chung (2017). We extracted all ≥50 bp regions with a sequencing depth of ≥4 median coverage and all ≥50 bp regions with ≥4 average sequence variation (positions with secondary base ≥5% coverage).*”

Overall, the new low-confidence region criteria suggested by reviewer confirms the quality of our MAGs which we already checked by polymorphism detection in order to identify potentially chimeric assemblies as shown in Supplementary Figure 2.

We also would like to point out that the MAG approach is widely used in state of the art *Wolbachia* studies. This includes the *D. melanogaster Wolbachia* comparative genomics seminal paper (Richardson et al. 2012 Plos Genetics) and more recently in similar studies tackling *D. simulans* (Signor 2017 Sci Rep), wRi (Turelli et al. 2018 Curr Biology), and *Onchocerca* (Choi et al. 2016 Nature Microbiology). Compared to these articles we have employed more stringent quality control criteria. However, to take into account for reviewer’s issues and to emphasize MAG-quality and potential confounding effects, we added the sentence: *“...However, because we found a certain level of genetic heterogeneity, we cannot completely exclude confounding factors from multiple Wolbachia infections in some of the MAGs.”*

**Reviewer #3** (Remarks to the Author):

The authors have made significant progress in advancing the state of the manuscript. I still have a few concerns that were not satisfactorily addressed in these revisions but should not prove problematic to the authors.

In the response to Reviewer #2 the authors state: “We followed the reviewer’s advice and no longer use the term “high quality” for our selected genomes that fulfilled our quality requirements.” Yet, the term high-quality still continues to be appear in parts of the manuscript – mainly Figure 1B – high-quality vs. core-quality.

Thanks for pointing out that we missed the replacing of “high quality” in Figure 1B. Now, it has been replaced by "MAGs passed QC" and "MAGs low quality"

This continues as issue that was articulated by Reviewer #2, but I had (and am still having) is the divide between mapping and de novo methods. In my understanding: “Mapping” is used to determine which samples have Wolbachia infection. Then de novo assembly occurs. And then “genome bins” are formed by “mapping” contigs against the 43 reference genomes.

Yes, in this way “mapping” correctly describes our procedure.

Then there is metric to determine a positive match. Somewhere, between Ln 445-499 there should be a clear definition of (formerly) high-quality vs core-quality, as the significance of these meanings is still unclear.

To clarify the quality based sample selection, we added the following sentences to the text: "*1005 MAGs passed our quality control criteria as defined below. Since some MAGs of lower quality are derived from exceptional hosts, for reconstructing the phylogenetic core tree, we used all 1161 MAGs that contain at least 33% core genes (Supplementary Table 3). 54 MAGs are rejected as not fulfilling any quality criteria.*"

I am in agreement with Reviewer #2 that this method leaves open a wide gap for co-infection, chimeric assemblies to persist in the data (which is okay for this type of dataset). This is supported by both the polymorphic rate (as reported by the authors) and the need in Ln 521-522 for the authors to select one representative when there are multiple copies of a core gene. The authors have not identified these core genes as single copy, but in practice many of the gene seem to be and a threshold should be applied that the core genes are believed to be single copy in the genome. The co-occurrence of core genes in a single MAG is generally referred to as "strain heterogeneity" in tools like CheckM and represent incorporation of multiple populations into a single MAG. Instead of selecting a representative of these core genes, which if incongruent with the other genes in the phylogenomic analysis will lead to longer branch lengths, the authors should exclude duplicate core genes in the alignment.

We agree that excluding these duplicated core genes is a good idea, even though the fraction of our multiple hits is very low (1.23%).
We therefore re-mapped our MAGs against the 316 core gene set and investigated in detail the multicopy cases. We observed 1.23 percent multi-copy hits which mainly are affected by three core genes, see figure.

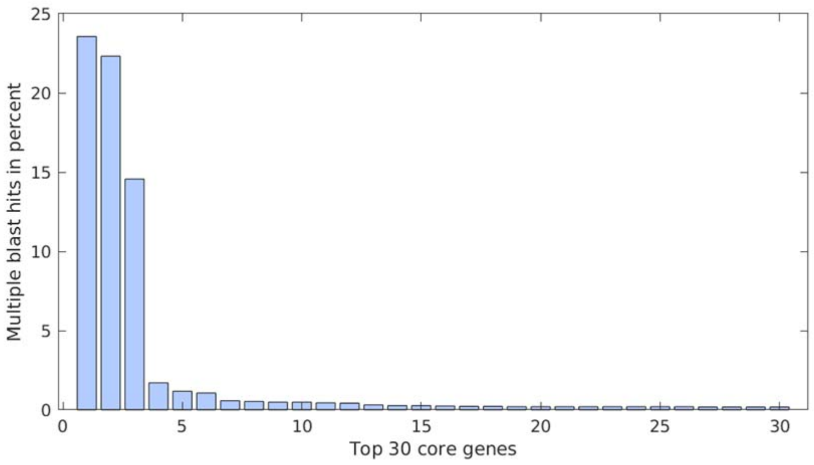


We excluded completely the top three core genes (rnhA, group_1162, group_1431) which show frequent multicopy cases and for other genes, we only selected the single hits.
We recreated the *Wolbachia* phylogenetic tree, following the identical previous procedure of aligning and concatenating core genes; we then generated a new RAxML tree. Five MAGs

are rejected based on the new tree criterion, reducing the total number to 1161 MAGs present in the core tree.

We replaced Figure 2a and the corresponding Supplementary File 1 with the new clean (multi-copy free) tree version, and added the main text "*To identify single copy genes, we excluded three core genes that show multiple hits in more than ten percent of our assembled genomes. For all remaining 313 core genes, we selected only gene sequences that are present as a single hit.*"

Indeed, a higher detailed structure in several branches and some higher bootstrap supports in the new tree suggest that the single copy filtering approach was helpful. We thank reviewer for this suggestion.  At the global scale, after collapsing branches, we observed some swops of closely related host branches, but the main tree structure and our conclusions remain consistent with both tree versions.

Our detailed host species specific analyses (Figure 4) are based on full genome MUMmer alignments, and hence are not affected by the multi-copy core gene issue.

I think the inclusion of polymorphic rate as a measure of population diversity is useful, overall, the authors could add emphasis of the role the population plays in the final dataset.

We added a sentence "*A high polymorphic rate can indicate potential incorporation of multiple populations into a single MAG or incorporation of host sequence of lateral gene transfer (LGT) events.*"
And to address a similar reviewer 2 issue we added somewhere else: "*However, because we found a certain level of genetic heterogeneity, we cannot completely exclude confounding factors from multiple Wolbachia infections in some of the MAGs.*"

I do not think that the amended methodology Ln 486-498 is sufficient to satisfy this concern. (1) Why only consider contigs >10kb, when contigs >1kb are included in the MAGs and shorter contigs may be the most problematic in terms of contamination?

Short genome regions >1kb may map unspecifically to different organisms: to *Wolbachia* as well to other bacteria. Indeed, most of the *Wolbachia* well curated reference genomes contain short <10kb regions that map to our 100,000 bacteria NCBI reference genomes. We therefore considered the threshold of >10kb reasonable to identify clearly contaminated regions than cannot be explained as non-specific matches. We hope this is acceptable for the reviewer

(2) The threshold of 97% nucleotide identity over 10kb is too strict for the identification of genetic material for potentially novel species (95% NAID) or genera (90% NAID). The authors should have a much looser cutoff here, with the goal of removing any possible contamination, airing on the side of false positives to ensure quality. Any %identity match at 10kb above 85%NAID to an organism other than Wolbachia would be problematic and should be removed. An alternative method to this BLAST approach would be to compare all

sufficiently large contigs (>10kb) against the reference database using FastANI and removing any non-Wolbachia matches.

We re-run our BLAST mapping using reviewer recommended thresholds >10kb and >85%NAID. We got more hits of reference species (Acetobacter tropicalis/ pomorum/ pasteurianus/ senegalensis/ tropicalis and Komagataeibacter xylinus E25) but not more contaminated genomes. All hits refer to the same previously detected contig SRR1183652_239 (*Drosophila melanogaster* related *Wolbachia* strain, already marked as low quality).
We also ran the BLAST mapping on our lower quality genomes and detected two additional contaminants. In total we have three contaminated contigs, but none of those contig-genes are involved in our core gene tree.
SRR1183652_239 (g_Acetobacter) host: Drosophila melanogaster
ERR969522_65 (g_Bartonella) host: Melophagus ovinus (Fly)
SRR2043491_8 (g_Rickettsia) host: Diachasma alloeum (Wasp)

Overall, the number of newly detected contaminants using reviewer's suggested setting is very low. We are remove the new contaminated contigs from our MAGs and are uploading an updated version to EBI.

Typos and clarifications:
Throughout: Bowtie2 is reference multiple times, each with a different spelling (e.g., BowTie2, bowtie2, etc.). Please unify with the correct spelling.

Thanks, we have fixed it by using only "*Bowtie2*" throughout the text.

Ln 210. "hoverlies" should be "hoverflies"

We corrected this typo.

Ln 213. The authors should still indicate their uncertainty by changing "this strain indicates a" to "this strain likely indicates a"

We agree and have added "likely".

Ln 258. "recon" should be "recognize"

We corrected this mistake.

Ln 446. "the 1220 Wolbachia-positive samples". Should be clarified as stated in Ln 415-417, these are specifically samples with >4X coverage.

We added "*having a coverage of at least 4X*".

REVIEWERS' COMMENTS:

Reviewer #1 (Remarks to the Author):

I have no further comments to the authors, who have addressed all issues to a very satisfying standard. I think the finding of Wolbachia rates correlating well with nuclear rates, but not with mitochondrial ones is a very cool additional outcome. Congrats again to the authors to an important piece of work that will certainly be a useful resource for the Wolbachia community and a starting point for many more analyses to come.

Reviewer #2 (Remarks to the Author):

I thank the authors for their significant additional work addressing my concern regarding contamination and LGTs in their MAG analyses. I am satisfied with their current analyses.

Reviewer #3 (Remarks to the Author):

I am excited to see the response of the community. The manuscript is well constructed and the work speaks for itself.
